# MyoRep: A Novel Reporter System to Detect Early Muscle Atrophy In Vitro and In Vivo

**DOI:** 10.1002/jcsm.70296

**Published:** 2026-05-12

**Authors:** Andrea D. Re Cecconi, Nicoletta Rizzi, Mara Barone, Federica Palo, Martina Lunardi, Mara Forti, Adriana Maggi, Paolo Ciana, Giulia Terribile, Michela Chiappa, Lorena Zentilin, Rosanna Piccirillo

**Affiliations:** ^1^ Department of Neuroscience Mario Negri Institute for Pharmacological Research IRCCS Milan Italy; ^2^ Direzione Servizi per la Ricerca—Settore Animal Care Unit University of Milan Milan Italy; ^3^ Department of Health Sciences University of Milan Milan Italy; ^4^ Molecular Medicine International Centre for Genetic Engineering and Biotechnology Trieste Italy

**Keywords:** cancer cachexia, in vivo imaging, muscle atrophy, reporter mouse

## Abstract

**Background:**

Muscle atrophy occurs during physiological (i.e., fasting) and pathological conditions (i.e., cancer) and anticipates death. Since not all patients will undergo muscle wasting, it would be highly useful to identify them soon to intervene early. We aim to generate a reporter system to follow only pathological, but not physiological, muscle wasting through in vivo imaging.

**Methods:**

Comparing the upstream non‐coding regions of a subset of atrophy‐related genes or atrogenes, using the *MuRF1* promoter as a backbone, we cloned various promoters upstream of *Firefly Luciferase*. The best hits selected in vitro were further compared in in vivo imaging if able to sense early atrophy induced by MCG101 sarcoma or sciatic nerve resection through plasmid electroporation or AAV9 injections. The best promoter was used to generate the reporter mouse MyoRep, expressing the cassette in all skeletal and cardiac muscles using the loxP system.

**Results:**

Luciferase assays showed that only the newly generated promoters of MuRF1, one containing glucocorticoid‐responsive elements or GRE (TWIST) (*p* ≤ 0.01, 1.7 FC) and a GRE‐less promoter (GREDEL) (*p* ≤ 0.0001, 1.6 FC), discriminated the supernatants from cachectic tumoural cells (C26) from non‐cachectic ones (4T1). Comparing both reporters electroporated in leg muscles, we found that GREDEL, but not TWIST, anticipated atrophy by 6 days in MCG101 carriers (*p* ≤ 0.05) and by 8 days upon denervation (*p* ≤ 0.05), recapitulating MuRF1 inductions. TWIST, but not GREDEL, drove an undesirable bioluminescent signal in vitro to dexamethasone (*p* ≤ 0.001, 1.5 FC) and in vivo upon fasting (*p* = 0.0553, 3 FC). GREDEL‐carrying AAV9 injected in the legs of Apc^Min/+^ mice unraveled sex‐different cachexia and anticipated body emaciation by 1 week (*p* ≤ 0.001, 3.7 FC). GREDEL was then used to generate the MyoRep mouse. Dorsal view of bioluminescent signal of MCG101‐carrying MyoRep mice increased already 6 days from tumour injection (*p* ≤ 0.01, 1.7 FC) when tumour is still unpalpable. Denervated MyoRep mice emitted a signal already 1 day after surgery (*p* ≤ 0.05, 1.4 FC), anticipating atrophy. Male Apc^Min/+^ mice display less musclin in their muscles (*p* ≤ 0.05, 0.4 FC) and plasma (*p* ≤ 0.01, 0.6 FC). Such mice, when expressing MyoRep in their muscle legs, were given the anti‐catabolic myokine musclin. The emitted signal was decreased by 30% 3 weeks after musclin‐AAV9 administration (*p* ≤ 0.05), supporting MyoRep useful to test anti‐atrophic drugs.

**Conclusions:**

Since MyoRep detects only pathological atrophy anticipating wasting, it represents an unprecedented tool to predict it early in diseases with local or systemic atrophy. It could also be useful to identify early biomarkers of atrophy and new drugs at once.

**Abbreviations**
AAV9: adeno‐associated virus 9
ANOVA: analysis of variance
APC: adenomatous polyposis coli
AUC: area under the curve
BCA: bicinchoninic acid
BSA: bovine serum albumin
BWL: body weight loss
C26: colon adenocarcinoma 26
CsCl: caesium chloride
Dexa: dexamethasone
DMEM: Dulbecco's Modified Eagle's Medium
EDTA: ethylenediaminetetraacetic acid
ELISA: enzyme‐linked immunosorbent assay
FBS: foetal bovine serum
FLUC: Firefly Luciferase
FoXO3: forkhead box O 3
GAS: gastrocnemius
GRE: glucocorticoid‐responsive elements
GUSB: β‐glucuronidase
HBSS: Hanks' balanced salt solution
IL‐6: interleukin‐6
Ipo8: Importin 8
kb: kilobase
MCG101: methylcholanthrene‐induced sarcoma 101
MuRF1: muscle RING finger 1
NF‐kB: nuclear factor kappa B
PBS: phosphate‐buffered saline
PCR: polymerase chain reaction
PEG: polyethylene glycol
PLB: passive lysis buffer
qPCR: quantitative polymerase chain reaction
RLUC: Renilla Luciferase
ROI: region of interest
SDS: sodium dodecyl sulphate
SEM: standard error of the mean
Smad 2/3: small mother against decapentaplegic 2/3
STAT3: signal transducer and activator of transcription 3
TA: tibialis anterior
TBP: TATA‐binding protein
VG: vector genome
WT: wild‐type

## 1 Introduction

Skeletal muscle atrophy consists of the progressive loss of muscle proteins due to an imbalance between protein generation and degradation. Atrophy occurs physiologically under certain circumstances as upon bed rest, fasting or following circadian rhythms. To a major extent, patients can face progressive muscle wasting during local or systemic diseases, such as loss of innervation or cancer, respectively. Other diseases causing severe muscle wasting are spinal cord injury with subsequent paralysis and disuse atrophy, amyotrophic lateral sclerosis, burn injury, heart or kidney failure, sepsis and muscular dystrophies. Muscle wasting is one of the hallmarks of cachexia (a debilitating condition involving involution of multiple tissues). Moreover, it is a major medical need since it reduces the response to therapies and leads to premature death, which usually arises when lean muscle mass loss reaches 30%–40% of body weight [1]. The overall prevalence of cachexia is quite high and increasing in industrialized countries. It is estimated that cachexia affects about 9 million patients, which is 1% of all patients with any disease [2]. Paradoxically, despite skeletal muscle being among the most abundant tissues in our body, it is one of the most ‘undrugged’ tissues, for which less drugs exist to repair or spare it. Either during physiological and pathological atrophies, the expression of a common set of genes, namely, ‘atrogenes’, is changed in muscles [3]. Among the genes that are early upregulated in various types of atrophy, there are those encoding for transcription factors, such as FoxO3, and muscle‐specific ubiquitin ligases, such as atrogin‐1 and MuRF1 [4, 5, 6], which promote muscle protein degradation through the proteasome.

The main goal of this project was to generate a reporter mouse able to emit a bioluminescent signal under a muscle‐specific promoter to detect early only pathological atrophy and not physiological one. This technology could also serve to identify early biomarkers of muscle atrophy for which there is poor knowledge. Over the years, we have analysed either binding sites for specific transcription factors driving atrophy (such as FoXO3, NF‐kβ [7], STAT3 [8], and Smad 2/3 [9]) or promoters of atrogin‐1 or MuRF1 in in vitro luciferase‐based assays to identify the best reporter promoter. Even if other reporter mice have been generated in the past as able to sense and signal muscle wasting [10, 11], they all failed to distinguish pathological atrophy from physiological one and to emit a signal highly anticipating atrophy. We believe that ours, namely, MyoRep (i.e., Myo standing for muscles and Rep for reporter), based on a much more improved version of the MuRF1 promoter, is better for the following two main reasons. Firstly, MyoRep is able to sense only pathological atrophy (caused by cut of the sciatic nerve or cancer) and not physiological one (as that induced by food deprivation), because such promoter has been deprived of glucocorticoid‐responsive elements (GRE). Secondly, its precocity in sensing atrophy has been obtained also by repeating in tandem the binding sites for specific transcription factors (such as TWIST [12], FoXO3 and myogenin‐binding sites [13]), highly reinforcing the responsiveness of such newly engineered promoter.

To assess the robustness of MyoRep promoter, we have evaluated its activity in in vitro luciferase assays, demonstrating its ability to drive the expression of a reporter gene (*Firefly Luciferase*) prior to protein loss. Additionally, MyoRep functionality has been confirmed in in vivo experiments. By transfecting such reporter plasmids in the muscles of adult mice, or by locally injecting AAV9 vectors carrying the MyoRep sequence upstream of *Firefly Luciferase* in leg muscles, we demonstrate its early activation and its ability to longitudinally track over time muscle wasting in mice by in vivo imaging. The generated MyoRep mouse expressing the cassette only in skeletal muscles and heart is able to emit a bioluminescent signal easily detectable by in vivo imaging upon muscle denervation or sarcoma MCG101 injection. This is in line with the 3R principle by minimizing animal use, reducing experimental variability and refining methodologies to obtain more physiologically relevant data through non‐invasive in vivo imaging [14].

Overall, we believe MyoRep mouse can be a useful tool to identify novel drugs against atrophy and/or useful early biomarkers or to better understand the dynamics behind muscle wasting and which muscles, among others, are preferentially lost during various diseases without the need of sacrificing many mice to weigh their muscles.

## 2 Material and Methods

### 2.1 Study Design

The sample size was determined using power analysis with G*Power, based on similar experiments previously published by our laboratory. Outliers were identified and removed using the robust regression followed by outlier identification (ROUT) test. All experiments were performed at least twice. The research objectives remained consistent across all experiments. For in vitro studies, we used cell cultures, while in vivo studies were made in mice. The overall experimental design included controlled laboratory experiments and in vivo imaging, with luciferase assays, qPCR and Western blotting used as major measurement techniques. Units were randomly assigned to different experimental groups, with mice randomized based on body weight. Blinding was not implemented in the in vivo experiments, as mice with subcutaneous tumours were easily distinguishable from those without.

### 2.2 Plasmids

MuRF1 promoter was cloned into the vector Pgl4.10[luc2] (Promega, Madison, WI) upstream of the *Firefly Luciferase* gene, with the cloning sites XhoI/EcoRV (Genescript, Piscataway, New Jersey, USA).

We used the following plasmids, all engineered by us:

- m(urine)MuRF1_pGL4.10[luc2], referred to as mMuRF1;
- mMuRF1‐TWIST_pGL4.10[luc2], referred to as TWIST;
- mMuRF1‐GREDEL_pGL4.10[luc2], referred to as GREDEL.

All the plasmids were co‐transfected with pRL‐TK‐Renilla Luciferase, used as an index of transfection efficiency to normalize the data (Promega, Madison, WI, USA) with a ratio of 1:50 (Renilla:Firefly).

### 2.3 Cell Transfection

Twenty‐four hours before transfection, C2C12 cells were seeded at 17 500 cell/cm^2^. We used 48 well‐plates for luciferase‐based assays. C2C12 cells were transfected using Lipofectamine 2000 (Invitrogen, Waltham, MA, USA), according to the manufacturer's instructions.

### 2.4 In Vivo Experiments

In vivo experiments have been made on C57BL/6J‐MyoRep mice and C57BL/6J‐Apc^Min/+^ and *wild‐type* littermates (The Jackson Laboratory—Charles River Italia, Calco, Italy). Four animals have been housed per cage in standard conditions with unlimited access to food and water, with 12 h of light and 12 h of dark. Inside the cage, environmental enrichment was provided. Males and females have been separated into different cages. Mice have been identified with a hole in different positions of the ears. Procedures involving animals and their care were conducted in conformity with institutional guidelines in compliance with national and international laws and policies. The Mario Negri Institute for Pharmacological Research IRCCS (IRFMN) adheres to the principles set out in the following laws, regulations and policies governing the care and use of laboratory animals: Italian Governing Law (D.lgs 26/2014; Authorization n° 19/2008‐A issued 6 March 2008 by Ministry of Health); Mario Negri Institutional Regulations and Policies providing internal authorization for persons conducting animal experiments (Quality Management System Certificate—UNI EN ISO 9001:2015—Reg. n° 6121); the National Institutes of Health (NIH) Guide for the Care and Use of Laboratory Animals (2011 edition); and European Union (EU) directives and guidelines (European Economic Community [EEC] Council Directive 2010/63/UE). The Statement of Compliance (Assurance) with the Public Health Service (PHS) Policy on Human Care and Use of Laboratory Animals has been recently reviewed and will expire in 2027 (Animal Welfare Assurance #929/2018PR—972/2020PR—157/2023PR—992/2023PR).

### 2.5 Statistical Analysis

For statistical analysis, data (means ± standard errors of the mean or SEMs) were analysed with GraphPad Prism 10.2 for Windows (Graph‐Pad Software, San Diego, CA, USA) with the following statistical tests: ordinary one‐way analysis of variance (ANOVA) for multiple comparisons followed by Tukey's or Dunnett's post hoc test, Kruskal–Wallis test followed by Dunn's post hoc test, two‐way ANOVA for multiple comparisons followed by Tukey's post hoc test, unpaired *t*‐test or Mann–Whitney test for comparisons of two groups, paired *t*‐test for paired analysis, Brown–Forsythe test for variance; *p ≤ 0.05; **p ≤ 0.01; ***p ≤ 0.001; ****p ≤ 0.0001. Where the asterisk(s) was/were omitted, the difference was nonsignificant (ns). The person in charge of the biostatistics is Andrea David Re Cecconi.

## 3 Results

### 3.1 In Vitro Tests to Identify the Best Reporter to Sense Myoblast Atrophy

Throughout the years, we have tested a long list of sequences both encoding for binding sites for transcription factors known to play a role in atrophy such as FoXO3, NF‐kβ [7], STAT3 [8], Smad 2/3 [9] or promoters of well‐known atrogenes (atrogin‐1 and MuRF1). Most of these sequences were provided in Firefly Luciferase reporter plasmids by collaborators (see Section 2) or engineered by us and listed in Table S1.

These vectors were transfected for 24 h in vitro in C2C12 myoblasts, and their possible response to various atrophic stimuli (supernatants of cancerous cells causing cachexia or not or HBSS to mimic starvation or dexamethasone or various cytokines) was measured to see if the reporter anticipated protein loss. Myoblasts were used instead of myotubes because the former are more easily transfectable with plasmids. A summary of all the experiments performed in vitro and in vivo is provided in Table S1. These experiments enabled us to understand that the MuRF1 promoter was among the best promoters to discriminate cachectic supernatant from non‐cachectic one, together with FoXO3 binding sites‐containing promoters (FHREDeltaXRE or Pgl4.10 2D4F) (Table S1). Since Pgl4.10 2D4F (cloned by us and containing 2 DBE and 4 FHRE upstream of *Firefly Luciferase*) was unable to signal early atrophy in electroporation studies in cancer‐bearing mice (Table S1), and a paper showed an interesting knock‐in of luciferase downstream of the endogenous MuRF1 promoter in rat [10], we decided to move on with the MuRF1 promoter by implementing it.

The original length of the MuRF1 promoter is 5 kb, too long to be used to generate a reporter in vivo. So, we generated the so‐called murine MuRF1 that is 628 bp long and was selected as the minimal one to signal atrophy in vitro (data not shown). Nonetheless, we generated two more variants from such mMuRF1 to test if TWIST transcription factor‐binding sites and GRE were needed or not to improve the earlier response to atrophy and its specificity. So, we compared in vitro the murine promoter of MuRF1 (or simply mMuRF1 that does not display changes to the genomic sequences), with mMuRF1 TWIST (or simply TWIST), and mMuRF1 TWIST GREDEL (or simply GREDEL) (their sequences are listed in Supporting Material and Methods). All these promoters were cloned upstream of *Firefly Luciferase*, and co‐transfection with a TK‐Renilla Luciferase vector served as control to normalize the data.

**FIGURE 1:**
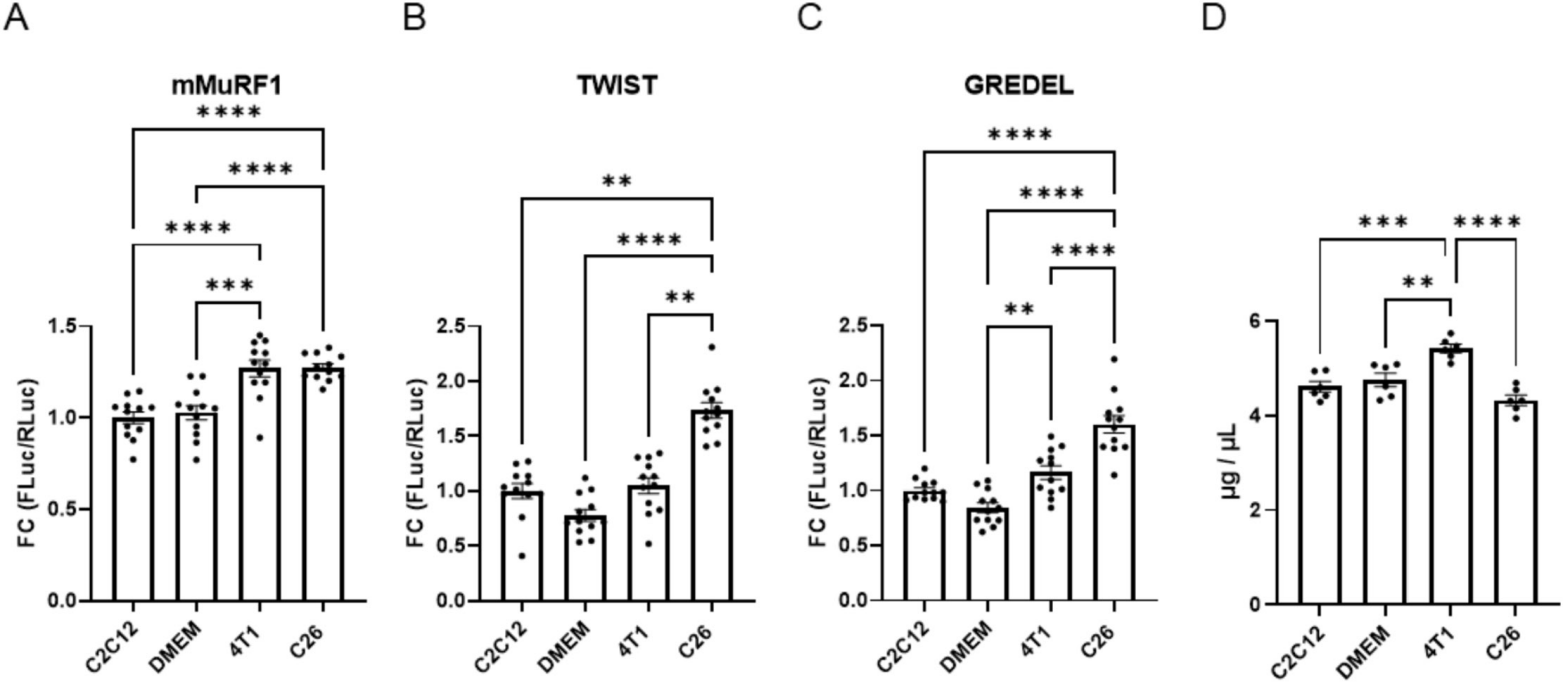
The variants TWIST and GREDEL of the murine promoter of MuRF1 (mMuRF1) can discriminate between media conditioned by cachectic from non‐cachectic cell lines, even before protein loss. Luciferase assays of C2C12 myoblasts transfected with mMuRF1 (A), TWIST (B) and GREDEL (C) reporter plasmids (all co‐transfected with pRL‐TK‐Renilla to normalize the data) for 24 h and treated with conditioned media from C2C12, 4T1 and C26 cell lines or DMEM as control for the next 24 h. One‐way ANOVA or Kruskal–Wallis test followed by Dunnett's or Dunn's post hoc test. **p ≤ 0.01, ***p ≤ 0.001, ****p ≤ 0.0001. *N* = 12. (D) Protein content of samples from Panels (A–C) was analysed by Bradford assay. One‐way ANOVA followed by Tukey's post hoc test. **p ≤ 0.01, ***p ≤ 0.001, ****p ≤ 0.0001. *N* = 6. All data are reported as mean ± SEM.

As an example, we transfected C2C12 myoblasts with each of these three vectors for 24 h and exposed them to media conditioned from C26 cells, as atrophying stimulus, or from 4T1 cells as a non‐cachectic one or from C2C12 cells and DMEM as controls for the next 24 h. We found that C26 supernatant induces the activity of Firefly Luciferase, measured in a luciferase‐based assay and normalized over Renilla Luciferase for all three vectors compared to C2C12 supernatant and DMEM. Importantly, only TWIST and GREDEL were able to discriminate between the non‐cachectic 4T1 and the cachectic C26, indicating that mMuRF1 promoter was less specific to the atrophic stimulus (Figure 1A–C).

To further test whether these vectors were able to signal atrophy before proteins diminished, we measured the protein content on the same samples whose luciferase activities are shown in Figure 1A–C. Aside from a slight protein accumulation in cells exposed to 4T1 conditioned medium (Figure 1D), our data indicate that overall, the variants TWIST and GREDEL of the murine promoter of MuRF1 can drive *Firefly Luciferase* expression upon cachectic stimulus, anticipating protein loss.

### 3.2 GREDEL Detects Systemic or Local Pathological Atrophy Better Than TWIST

**FIGURE 2:**
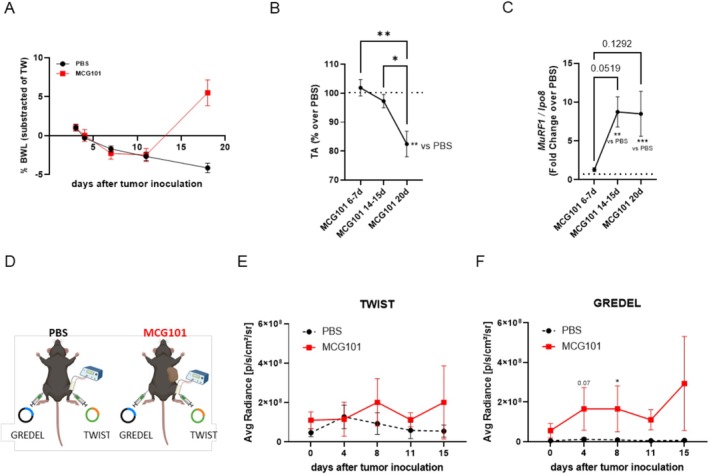
MCG101‐carrying mice display atrophy detectable earlier by GREDEL than TWIST reporter. (A) Percentage of body weight loss (BWL%) subtracted of tumour weight of male mice injected subcutaneously with PBS or MCG101 over time. Two‐way ANOVA followed by Tukey's post hoc test. ****p ≤ 0.0001. *N* = 5. (B) TA weight (% over PBS, dotted line) of MCG101 carriers sacrificed at different times. One‐way ANOVA followed by Tukey's post hoc test. *p ≤ 0.05, **p ≤ 0.01. MCG101 20 days vs. PBS, unpaired *t*‐test, **p ≤ 0.01. *N* = 7–33. (C) MuRF1 mRNA expression measured by qPCR in TA from mice of Panel (B). One‐way ANOVA followed by Tukey's post hoc test and unpaired *t*‐test. **p ≤ 0.01, ***p ≤ 0.001. (D) Graphical representation of the experimental scheme, made with BioRender. Analysis of photon emission of TA expressing TWIST (E) or GREDEL reporters (F) comparing the AUC of PBS and MCG101 hosts. (E) Unpaired *t*‐test for AUC, *p ≤ 0.05, *N* = 3–5; (F) unpaired *t*‐test for AUC, **p ≤ 0.01, *N* = 3–5. At Days 4 and 8 PBS vs. MCG101: *p ≤ 0.05, Mann–Whitney test. All data are reported as mean ± SEM.

We next compared the TWIST and GREDEL vectors by electroporating them in TA of sarcoma MCG101‐bearing mice as a model of cachexia for their ability to signal in vivo atrophy. This model shows body weight loss (BWL) 18 days after tumour injection compared to PBS‐injected mice (Figure 2A). When we sacrificed mice at 6–7, 14–15 and 20 days after tumour cell injection, we found that tumour weight was significantly greater at 14–15 and 20 days compared to 7 days (Figure S1). The weights of TA and gastrocnemius (GAS) were reduced in size only 20 days following tumour injection compared to 6 and 7 days and to PBS‐injected mice (Figures 2C and S2A). Instead, MuRF1 mRNA expression was already increased in TA at 14 and 15 days, anticipating muscle wasting (Figure 2C).

TWIST‐ and GREDEL‐expressing plasmids were electroporated in TA from MCG101‐ and PBS‐bearing mice, as shown in Figure 2D. When we analysed the photon emission of TA expressing TWIST or GREDEL reporters, comparing the area under the curve (AUC) of PBS and MCG101 hosts, we found a greater bioluminescence emission in MCG101‐bearing mice for both vectors within 15 days from tumour injection, before muscle atrophy (Figure 2E,F). Activation of both vectors was confirmed in TA from MCG101 carriers in ex vivo luciferase assay (Figure S2B). Notably, GREDEL, but not TWIST, was activated already 4 and 8 days after tumour injection in MCG101 hosts with respect to PBS ones (Figure 2F). TWIST showed greater variations in photon emissions with respect to GREDEL, when comparing the AUC of tumour‐free mice, possibly indicating that TWIST is more sensitive than GREDEL to circadian cycle‐related variations (Figure S2C). As further control, we measured the content of plasmids encoding for TWIST and GREDEL electroporated in vivo with ad hoc primers (those for measuring ampicillin resistance and another set to detect luciferase genes) on overall DNA extracted from electroporated muscles, and found comparable amounts of electroporated plasmids detected with both set of primers (Figure S3). In the attempt to understand if GREDEL was able to sense in vitro as in vivo MCG101 cells, we compared the ability of GREDEL‐transiently expressing C2C12 cells to increase bioluminescence 6, 24 and 48 h after exposure to media conditioned by MCG101 cells with that conditioned by C26 ones (Figure S4A–D). We found that both supernatants that caused protein loss only 48 h later (Figure S4D) increased GREDEL‐derived bioluminescence at all timepoints (Figure S4A–C), supporting GREDEL as an early sensor of muscle atrophy in vitro and in vivo.

**FIGURE 3:**
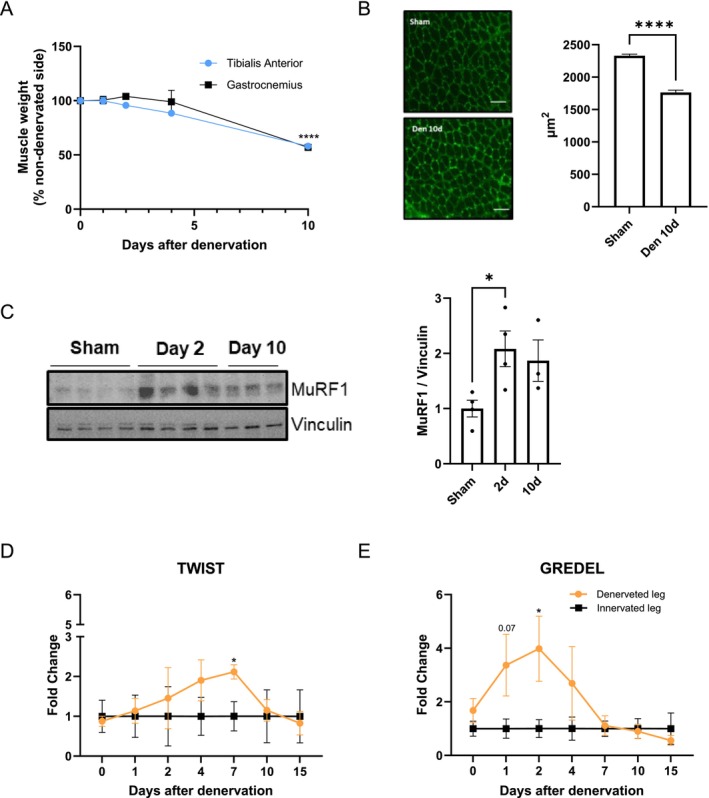
GREDEL reporter detects earlier atrophy also in mice subjected to denervation. (A) Gastrocnemius (GAS) and tibialis anterior (TA) weight (% of non‐denervated side) of denervated leg at different times. One‐way ANOVA test followed by Dunnett's post hoc test. ****p ≤ 0.0001. *N* = 3–26. (B) Representative images and relative quantitations are shown for transverse sections of TA fibres stained with wheat germ agglutinin (WGA) 10 days after denervation. Mean ± SEM. Scale bar: 50 μm. At least 622 fibres per group were analysed. Mann–Whitney test, ****p ≤ 0.0001. (C) WB for MuRF1 of TA of sham‐operated leg and denervated leg at Days 3 and 10 after surgery. Vinculin is used as loading control. Unpaired *t*‐test. *p ≤ 0.05. *N* = 3–4. Analysis of photon emission of TA expressing TWIST (D) or GREDEL reporters (**E**) upon denervation. Fold change of denervated leg over innervated one. (D) Two‐way ANOVA followed by Tukey's post hoc test. *p ≤ 0.05. *N* = 4. (E) Two‐way ANOVA followed by Tukey's post hoc test. Unpaired *t*‐test for Days 1 and 2. *p ≤ 0.05. *N* = 4. All data are reported as mean ± SEM.

To validate the ability of TWIST and GREDEL reporters to detect local muscle atrophy, we further tested them in a mouse model where cut of the sciatic nerve induces atrophy of GAS and TA muscles [15]. The denervated leg showed a loss of TA by about 10% (*p* = 0.07) only 4 days after surgery and both TA and GAS by 40% 10 days after denervation, compared to the sham‐operated leg (Figure 3A). CSA was decreased accordingly (Figure 3B). Interestingly, protein levels of MuRF1 were increased already 2 days after surgery in denervated TA (Figure 3C), anticipating muscle atrophy, as shown by others in [4]. To assess the bioluminescent signal, each mouse was electroporated 2 weeks before denervation in each TAs with either TWIST‐ or GREDEL‐encoding plasmids, and the denervated leg was compared to the sham‐operated one. Interestingly, while the TWIST reporter got activated 7 days after denervation (Figure 3D), GREDEL was able to drive Firefly Luciferase activity as early as 1–2 days in the denervated leg compared to the sham‐operated one (Figure 3E), anticipating muscle mass loss.

### 3.3 TWIST Is Undesirably Sensitive to In Vivo Physiological Atrophy

Glucocorticoids are known to induce muscle atrophy both in vitro and in vivo [16]. They contribute to muscle atrophy in cancer cachexia [17] and denervation [18], but they are also released from the hypothalamic‐pituitary‐adrenal axis during fasting in mice [19]. Moreover, corticosterone and cortisol are secreted in a circadian‐dependent manner in both animals and humans [20, 21], being at the intersection between pathological and physiological atrophies.

Anorexia occurs also during cancer cachexia, but we aimed to generate a reporter mouse sensitive only to cachexia and not to the possibly reduced food intake. Even if MCG101 hosts eat as well as PBS mice (Figure S5), other cachectic tumours also cause anorexia in mice, such as C26 [22]. These considerations prompted us to compare TWIST and GREDEL reporters also in response to physiological atrophies induced by circadian rhythms or fasting. The best reporter to pathological atrophies should be insensitive to either of them.

**FIGURE 4:**
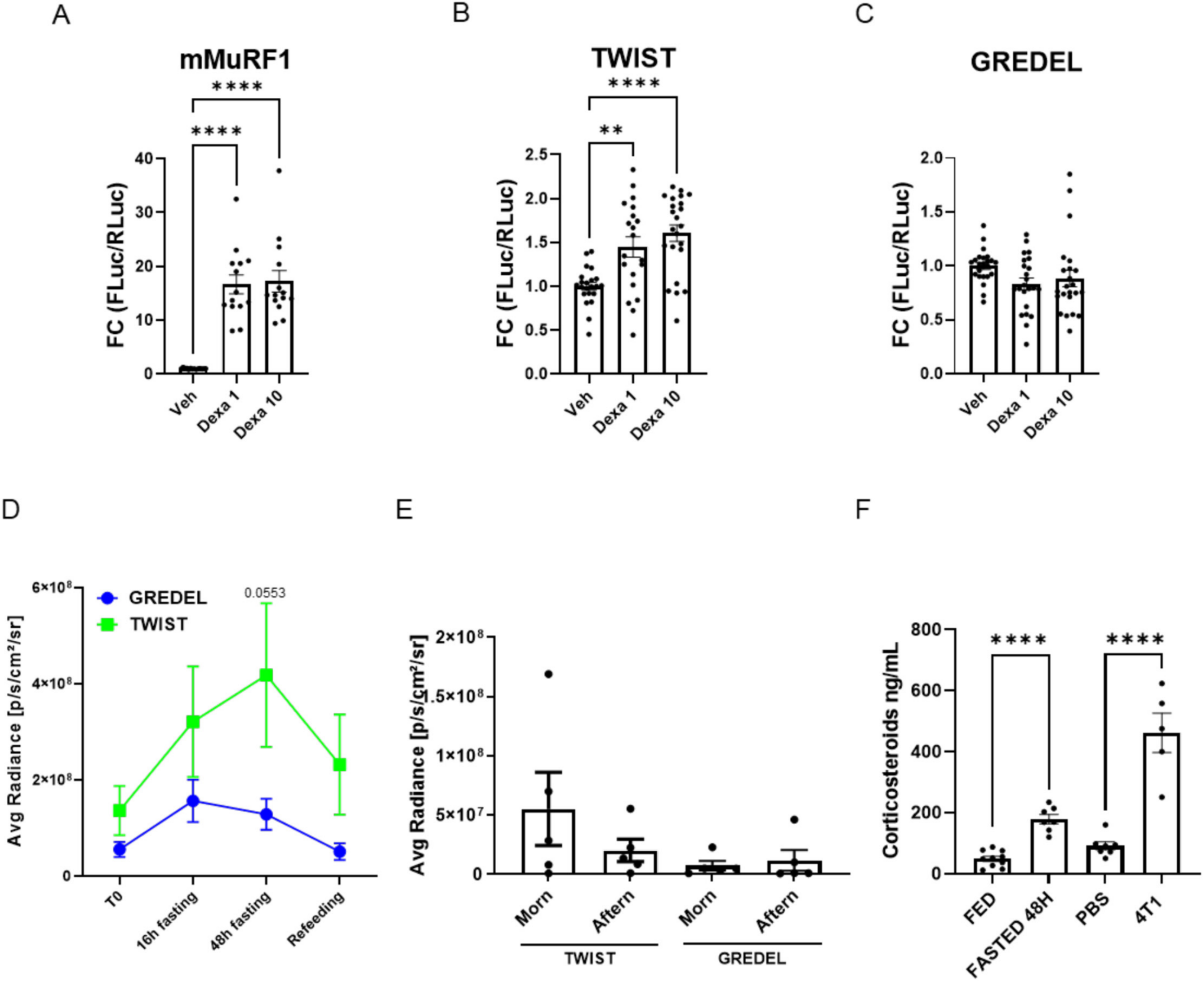
GREDEL reporter is not sensitive to circadian rhythm variations as TWIST. Luciferase assay analysis of C2C12 myoblasts transfected with mMuRF1 (A), TWIST (B) or GREDEL (C) reporter plasmids and treated with 1‐ or 10‐μM dexamethasone (Dexa 1 or 10, respectively). Kruskal–Wallis test followed by Dunn's post hoc test. **p ≤ 0.01, ****p ≤ 0.0001. *N* = 14–23. (D) Analysis of photon emission comparing area under the curve (AUC) of TA expressing GREDEL reporter with TA expressing TWIST reporter. Unpaired *t*‐test for AUC, *p* = 0.0553. *N* = 12. (E) Analysis of photon emission comparing TA expressing GREDEL with TA expressing TWIST in the morning and in the afternoon of tumour‐free mice. One‐way ANOVA and Brown–Forsythe test, nonsignificant. *N* = 5. (F) ELISA for corticosteroids in the plasma of mice fed, fasted or injected with PBS or 4T1 cells for 21 days. Unpaired *t*‐test, ****p ≤ 0.0001, *N* = 5–10. All data are reported as mean ± SEM.

Firstly, we confirmed in luciferase assays that only C2C12 transfected with mMuRF1 and to a lesser extent with TWIST, but not with GREDEL reporter plasmids, responded to 1‐ or 10‐μM dexamethasone (Dexa) (Figure 4A–C). Secondly, when we electroporated these vectors in TA of mice subjected to 48 h of fasting, unlike GREDEL, TWIST tended to be activated (Figure 4D), and its emission reduced upon refeeding, confirming its undesirably sensitivity to physiological atrophy. Additionally, despite not being significant, GREDEL exhibited a trend toward lower variability than TWIST in the bioluminescence signal detected in TA of healthy mice between morning and afternoon (Figure 4E), in accordance with the data shown in Figure S2C.

The activation of TWIST in response to fasting may be due to corticosteroids, as we observed higher plasma levels of corticosteroids in mice fasted for 48 h compared to fed mice (Figure 4F). Of note, corticosteroid levels were also found elevated in plasma from 4T1‐bearing mice compared to PBS‐injected controls (Figure 4F). 4T1 is a triple negative breast cancer cell line that does not cause cachexia in vivo [23]. Altogether, these data prompted us to choose the GREDEL promoter to generate either AAV9 or the reporter mouse and to rename it MyoRep, where Myo stands for muscle and Rep for reporter.

### 3.4 MyoRep Discriminates Sex‐Specific Muscle Atrophy in Cachectic Apc^Min/+^ Mice

Then, we generated an AAV9 expressing MyoRep upstream of *Firefly Luciferase*. To further validate its functionality during cancer cachexia, we injected MyoRep‐AAV9 into the TA muscle of Apc^Min/+^ and WT mice of both sexes at 8 weeks of age. We then monitored the mice using in vivo imaging until 18 and 19 weeks of age, when muscle atrophy becomes evident [24]. The Apc^Min/+^ mouse model develops spontaneously intestinal tumours and cachexia, mimicking colorectal cancer‐associated muscle wasting with males more severely affected than females [24].

**FIGURE 5:**
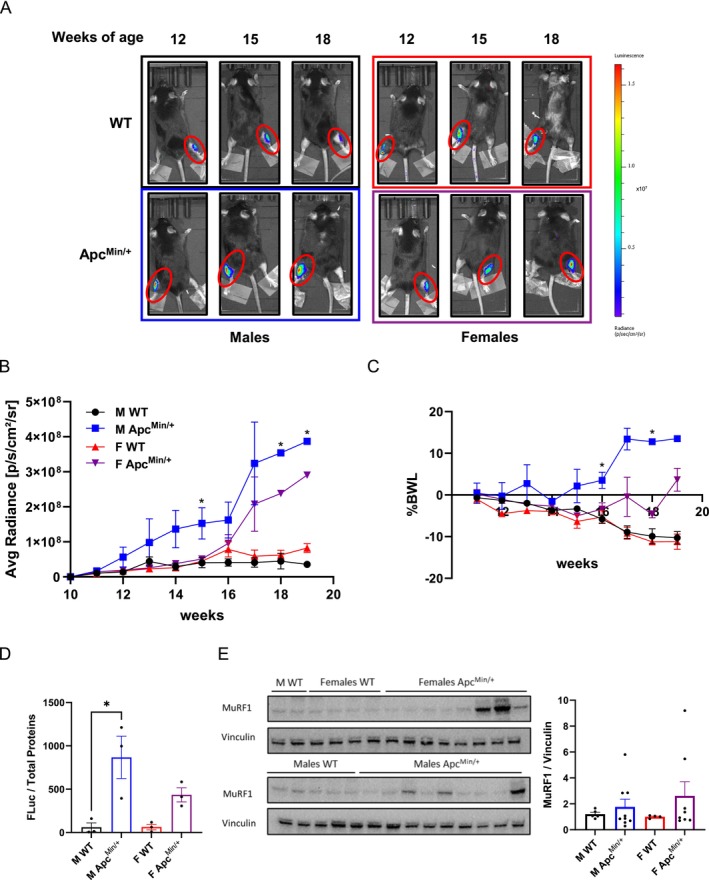
GREDEL reporter can detect the sex‐specific atrophy of cachectic Apc^Min/+^ mice. (A) Representative images of C57BL/6 WT and Apc^Min/+^ mice of both sexes acquired by in vivo imaging. One TA injected at 8 weeks of age with 10^12^ vg/mL of MyoRep‐carrying AAV9, the contralateral leg injected with PBS. (B) Analysis of photon emission from the region of interest over time (ROI on TA). Two‐way ANOVA, Tukey's post hoc test, *p ≤ 0.05, *N* = 4. (C) Percentage of BWL of C57BL/6 WT and Apc^Min/+^ mice over time. Two‐way ANOVA followed by Tukey's post hoc test. *p ≤ 0.05. *N* = 5. (D) Luciferase assay analysis of TA from male and female WT and Apc^Min/+^ mice at 15 weeks of age, normalized on total protein content. One‐way ANOVA, Tukey's post hoc test, *p ≤ 0.05, *N* = 3. (E) Western blot for MuRF1 of TA from male and female WT and Apc^Min/+^ mice at 15 weeks of age. Vinculin as loading control. (F) Quantitation of western blot in Panel (E). *N* = 4–9. All data are reported as mean ± SEM.

Once a week, we imaged the mice under anaesthesia using the IVIS machine (PerkinElmer) and tracked the onset and progression of muscle atrophy by measuring the bioluminescent signal from the injected leg (Figure 5A). The region of interest (ROI) was quantitated using dedicated software, and the signal was plotted over time (Figure 5B). As expected, Apc^Min/+^ males exhibited a higher signal than age‐matched Apc^Min/+^ females, starting from 15 weeks of age (Figure 5B). Additionally, we weighed the mice weekly and plotted their body weights over time, further confirming the sex differences in BWL, starting from 16 weeks of age (Figure 5C). This means that MyoRep activation precedes BWL by 1 week in these cachectic mice. Interestingly, sex‐specific activation of MyoRep is also detected in TA collected at 15 weeks of age and analysed in ex vivo luciferase assays (Figure 5D), confirming the data obtained in vivo. Moreover, MyoRep analysis proved to be more sensitive and less variable in detecting muscle atrophy in vivo compared to measuring MuRF1 ex vivo by western blot in TA isolated from mice at 15 weeks of age (Figure 5E,F). In fact, the MyoRep vector does not contain just the MuRF1 promoter but also repeated and appropriately modified sequences of the MuRF1 promoter designed to enhance sensitivity to muscle atrophy while also removing GREs, as previously described.

These data further prompted us to use the GREDEL vector to generate the MyoRep reporter mouse, which expresses this vector throughout the skeletal muscles and heart (Figure S6). This makes it a valuable tool for studying virtually any disease with associated muscle atrophy and *MuRF1* induction.

### 3.5 The MyoRep Mouse Detects Early Systemic and Local Atrophy

**FIGURE 6:**
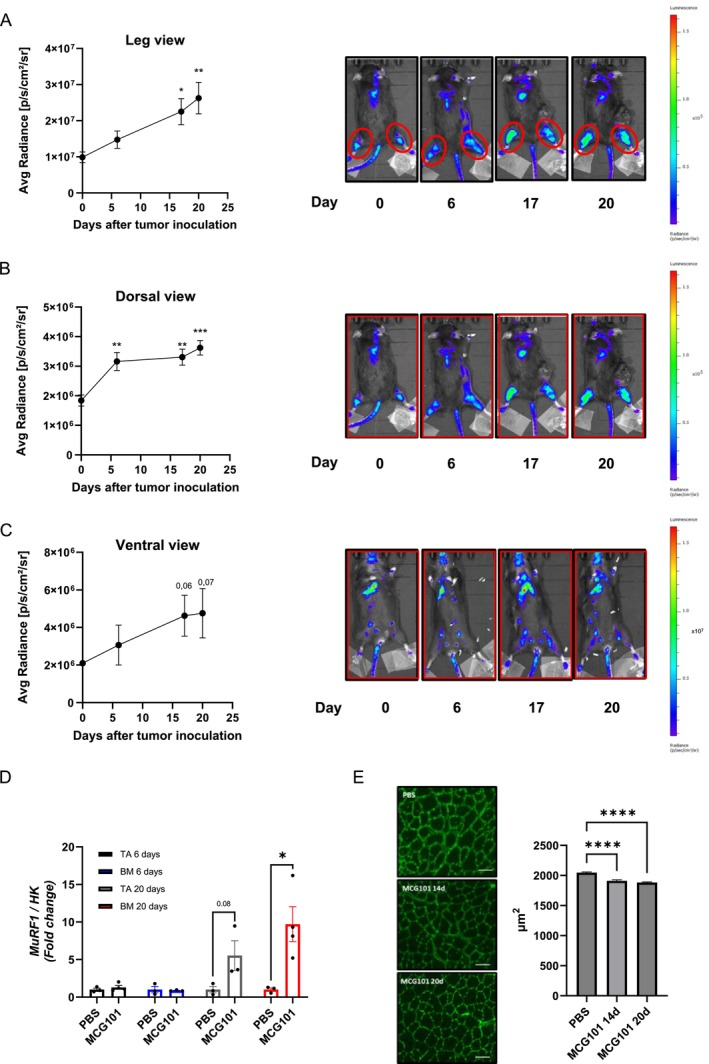
The dorsal scan of MCG101‐carrying MyoRep mice detects earlier atrophy than leg or ventral views. (A) Analysis of photon emission from the region of interest over time (ROI, leg in red) and representative images of MCG101 bearing‐MyoRep mice acquired by in vivo imaging. One‐way ANOVA, Dunnett's post hoc test vs. Day 0, *p ≤ 0.05, **p ≤ 0.01, *N* = 5. (B, C) Analysis of photon emission from the ROI in red on whole mouse on dorsal view (B) or ventral view (C) and representative images of MCG101 bearing‐MyoRep mice acquired by in vivo imaging. (B) One‐way ANOVA, Dunnett's post hoc test vs. Day 0, **p ≤ 0.01, ***p ≤ 0.001, *N* = 5. (C) One‐way ANOVA, Dunnett's post hoc test vs. Day 0, *N* = 5. (D) *MuRF1* expression measured by qPCR in TA and back muscle (BM) 6 and 20 days after tumour inoculation. HK = housekeeping gene for normalization (β‐glucuronidase, *Gusb* or TATA‐binding protein, *Tbp* or Importin 8, *Ipo8*). Unpaired *t*‐test. *p ≤ 0.05, *N* = 3–4. (E) Representative images and relative quantitations of transverse sections of TA muscle fibres stained with WGA are shown for MCG101‐bearing mice. Kruskal–Wallis test followed by Dunn's multiple comparisons test. ****p ≤ 0.0001. All data are reported as mean ± SEM.

To assess MyoRep's ability to detect muscle atrophy, we used the MCG101 mouse model of cancer cachexia, as previously shown in Figure 2. Following the subcutaneous injection of MCG101 cells, we monitored mice using in vivo imaging until Day 20, imaging them 6, 17 and 20 days and analysing photon emission from the ROI in the leg, dorsal and ventral views (Figure 6A–C).

The bioluminescence emission analysis showed that MyoRep was activated at Days 17 and 20 after tumour injection in the legs and ventral total view (Figure 6A,C), and as early as Day 6 in the dorsal total view (Figure 6B)—at times when muscle atrophy is not present yet (Figure 2C). This demonstrates the early activation of MyoRep that even anticipates increases in mRNA levels of *MuRF1*, as measured by qPCR in TA and back muscles, where *MuRF1* increases in the dissected tissues only 20 days post‐tumour injection (Figure 6D). Fourteen and 20 days after tumour injection, also CSA of TA were found decreased in MCG101 hosts (Figure 6E). Importantly, we imaged PBS‐injected MyoRep mice and those carrying a non‐cachectic tumour such as MC38 [25] and found negligible changes in photon emission from all three views (Figure S7A–C).

**FIGURE 7:**
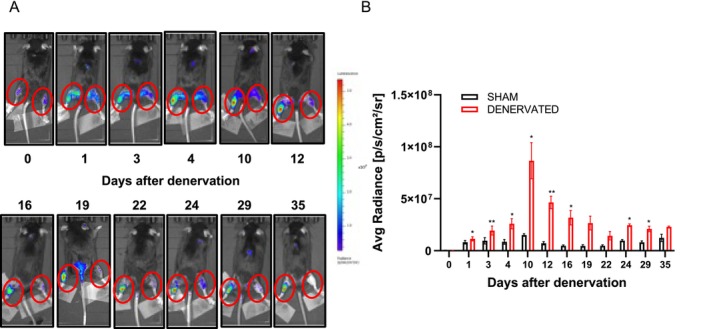
MyoRep mice subjected to denervation show increased bioluminescent signal from the denervated leg before muscle weight loss. (A) Representative images of MyoRep mice acquired by in vivo imaging at different times. The left leg is denervated, while the right one is sham‐operated. (B) Analysis of photon emission from the ROI (hindlimb muscles, red circle). Paired *t‐*test, *p ≤ 0.05; **p ≤ 0.01. *N* = 4. All data are reported as mean ± SEM.

To test if MyoRep transgenic mice detect also local atrophy, we denervated them as described in Figure 3 and performed in vivo imaging analysis from Day 1 post‐denervation until Day 35, as shown in Figure 7A. MyoRep mice subjected to denervation exhibited increased bioluminescence in the denervated leg compared to the sham‐operated one, even at Day 1 post‐denervation (Figure 7B), predicting the onset of muscle weight loss (Figure 3A). At 35 days after denervation, we sacrificed mice, collecting various tissues (muscles, spleen, brain, heart and liver), and we ex vivo imaged muscles and organs (Figure S8). Analysis of photon emission from dissected muscles from MyoRep mice, comparing the denervated leg with the sham‐operated contralateral one, revealed bioluminescence activation in the TA, GAS and soleus (Figure S8). As expected, no activation was observed in the quadriceps, as it was not affected by the cut of the sciatic nerve, nor in the other organs analysed.

### 3.6 The MyoRep Mouse Detects the Anti‐Atrophic Effects of Musclin

**FIGURE 8:**
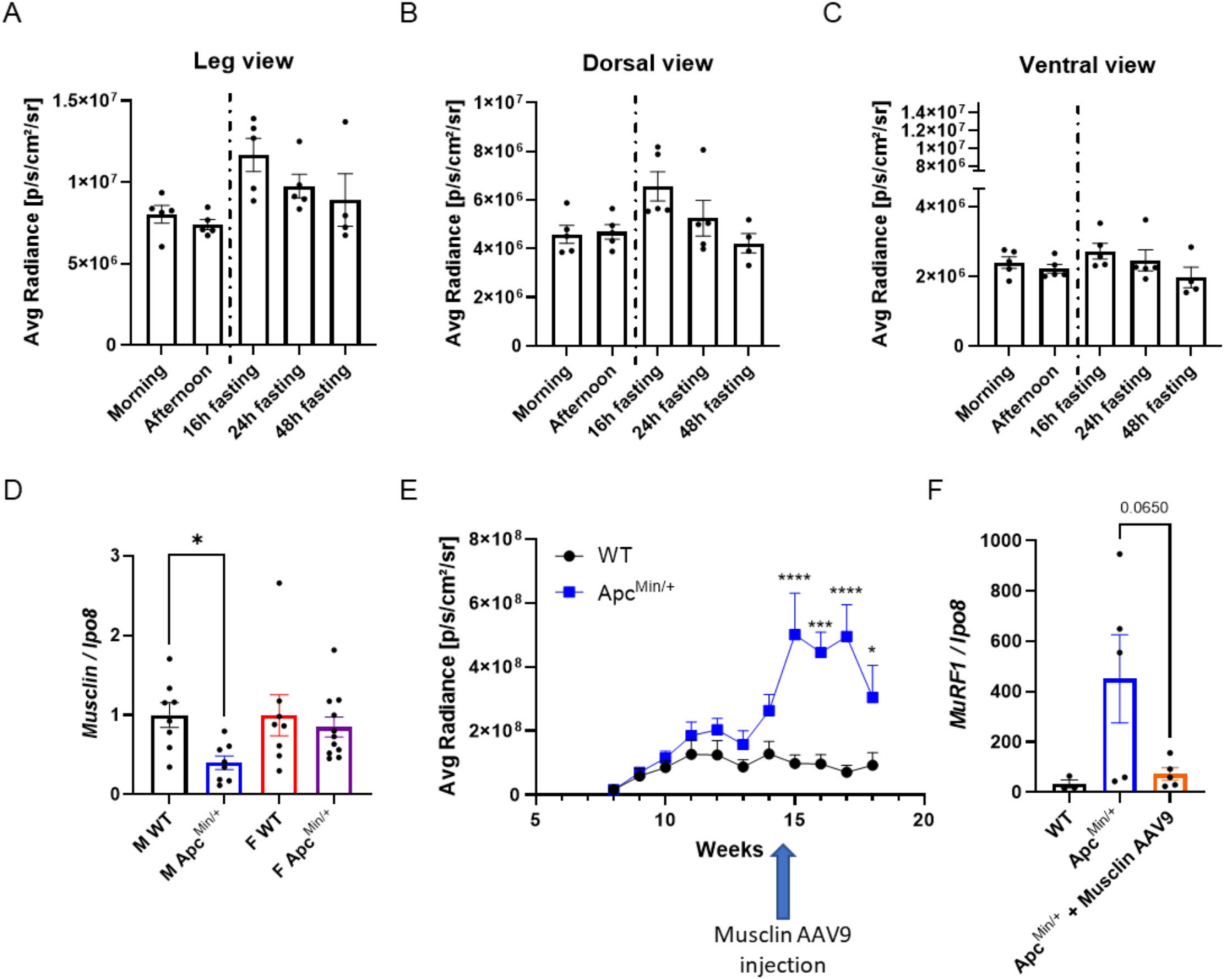
MyoRep mice do not display photon emission upon physiological atrophy and decrease tumor‐induced signal after musclin administration. (A–C) Analysis of photon emission from the ROI (legs in Panel A, whole mice in dorsal view in Panel B and in ventral view in Panel C) collected during various times of the day or with fasting of various time lengths of MyoRep mice. One‐way ANOVA, Tukey's post hoc test. *N* = 4–5. (D) The mRNA expression of *musclin* measured by qPCR in TA of WT and Apc^Min/+^ male and female mice at 12 weeks of age. *Ipo8*, as housekeeping gene. Unpaired *t*‐test or Mann–Whitney test. *N* = 7–13. *p ≤ 0.05. (E) Analysis of photon emission from the ROI on hindlimb leg over time. The arrow indicates the time of treatment in TA with musclin‐AAV9. Two‐way ANOVA, Tukey's post hoc test. *N* = 5–7. *p ≤ 0.05, ***p ≤ 0.001, ****p ≤ 0.0001. (F) The mRNA expression of *MuRF1* measured by qPCR in TA after musclin‐AAV9 injections. *Ipo8*, as housekeeping gene. Unpaired *t*‐test. *N* = 3–5. All data are reported as mean ± SEM.

As further control, we aimed to assess whether MyoRep mice were unresponsive to physiological muscle atrophy, as shown in Figure 4. We performed in vivo imaging in healthy MyoRep mice in the morning and afternoon, as well as after 16, 24 or 48 h of fasting, and observed no significant variations in photon emission across different ROIs (leg, dorsal and ventral views) (Figure 8A–C). Importantly, these results confirm that our reporter mouse is not affected by circadian rhythms or fasting, assuring its specificity for pathological muscle atrophy only.

Finally, in the attempt to test whether MyoRep activation could be reversed by a possible anti‐cachectic treatment, we measured musclin content in plasma and muscles of Apc^Min/+^ mice at various ages. Musclin is a myokine, in other words, a molecule released by muscles (in response to exercise), whose levels we previously found to be reduced in TA of C26‐bearing male mice. The electroporation of a plasmid encoding musclin in TA of C26 carriers partially preserved their fibre area [26]. Interestingly, we found decreased levels of musclin mRNA in TA only in Apc^Min/+^ males and not in Apc^Min/+^ females at 12 weeks of age (Figure 8D), and also in the plasma at 18 weeks of age only in males (Figure S9). So, we injected musclin‐expressing AAV9 into the TA of 14.5‐week‐old Apc^Min/+^ males and found that this treatment reduced photon emission at 18 weeks of age, restraining MyoRep activation (Figure 8E). In the same animals, we also measured MuRF1 levels in the TA at the time of sacrifice (18 weeks of age) to see whether musclin overexpression protected muscles from atrophy. We observed a trend toward reduced *MuRF1* mRNA levels, confirming that musclin acts as an anti‐catabolic myokine and supporting MyoRep as able to detect these changes in vivo before sacrifice (Figure 8F). Of note, the binding sites inserted in the MyoRep are well conserved across species from *Ovis aries* and *Sus scrofa domesticus* to *Homo sapiens* (analysis done using BLAST database; Figure S10), thus facilitating the application of MyoRep technology to other species.

These results highlight the potential of MyoRep to test drugs able to mitigate muscle atrophy, such as musclin.

## 4 Discussion

### 4.1 Advantages of MyoRep Technology Over Existing Reporter Tools

Muscle wasting is an unsolved medical issue that afflicts patients with very diverse chronic illnesses, causing premature death. To find novel in vivo tools to study muscle atrophy should be prioritized to accelerate the discovery of biomarkers predicting in advance atrophy and the findings of novel therapeutics to obviate this deleterious process. A challenge in the field is the absence of a tool that discriminates pathological atrophy from physiological one as that occurring far from a meal or following circadian rhythm fluctuations. We invented MyoRep technology to solve this issue. The model we created is based on reporter mouse technology which uniquely combines the presence of insulating sequences and a constitutively and ubiquitously open locus [27, 28]. Since the MyoRep mouse is the result of crossing the Luc mouse embedded with the stop codon under CRElox system with the ACTA‐Cre mouse, the progeny—that is, double‐transgenic—will express the MyoRep cassette under the promoter of actin that is equally expressed in different fibre‐types [29]. This is important because oxidative fibres are less prone to atrophy than glycolytic ones in different kinds of atrophy (as that induced by cancer).

MyoRep is able to sense early atrophy either in vitro or in vivo, so that it can be potentially used to identify early biomarkers of atrophy. Moreover, MyoRep can sense atrophy initiated either by local denervation or systemic cancer. These reporter mice are able to emit a local bioluminescent signal easily detectable by in vivo imaging upon muscle denervation already 1 day after the cut of the sciatic nerve, when muscles were not reduced in size yet, as also shown by Wei Li and coworkers [10] in a rat model where a reporter gene was knocked into an intron of the *MuRF1* gene. On top of that, MyoRep mice emit a signal that we found originating from back muscles earlier than other muscles when injected with a cachexia‐promoting tumour such as the sarcoma MCG101, indicating a preferential loss of back muscles over others. Instead, reporter cells locally injected reveal atrophy in nude mice bearing human pancreatic tumours, but only in a local way and with no precocity [30]. Similarly, the reporter mouse useful to monitor proteasome activity detects a late and not an early event in the atrophic process [11].

An important advantage of MyoRep mouse over existing reporter mice and systems is that it is insensitive to circadian rhythms. MyoRep mice are in fact insensitive to atrophy upon fasting or upon diurnal‐nocturnal variations of muscle size. This was obtained by deleting the GRE sequence by MuRF1 promoter. The other existing reporter systems still hold GRE in their regulatory sequences, making them unable to discriminate between pathological and physiological atrophy. For example, the reporter mouse, described above and generated by Wei Li and colleagues, is highly responsive to dexamethasone injections [10]. A common set of genes, namely, atrogenes, drives the atrophic processes in either physiological or pathological atrophies, making challenging the possibility of distinguishing between them. We believe that a GRE‐containing reporter mouse is not suitable for in vivo experiments aimed at identifying early biomarkers of atrophy or ad hoc drugs because it is more amenable to non‐specific and undesirable activation (as we also showed by higher basal TWIST‐based emissions in PBS‐injected mice). Indeed, we provide data indicating that glucocorticoid levels are increased in plasma of mice either upon fasting or injected with a non‐cachectic tumour (4T1, that we showed unable to cause atrophy and unable to increase plasma pro‐inflammatory IL‐6 cytokine [23]), further supporting their involvement in physiological atrophy as well as during tumour progression with no cachexia.

As other in vivo reporter systems to sense atrophy, MyoRep retains some advantages as the possibility to spare animals during experiments because each animal can be followed over time in longitudinal studies without the need to sacrifice mice at various timepoints to measure muscle size and atrogene expression. In the past, we have successfully used microCT to follow in vivo atrophy initiated by C26 tumours or ALS [31]. Unfortunately, microCT is more time‐consuming and labor‐intensive than in vivo imaging with MyoRep and does not allow to visualize in advance atrophy before its onset in mice. Nonetheless, both systems are in accordance with the 3R rule (replacement, refinement and reduction) as recommended by [14, 32, 33].

### 4.2 Future Applications for the MyoRep Technology

Better than other approaches, the MyoRep tool may serve not only to identify early biomarkers of atrophy in plasma and muscle, but also to better understand the dynamics of involved muscles (which muscles are more prone to different kinds of pathological atrophy, as revealed by in vivo imaging). We will cross MyoRep mouse with other disease models as SOD1G93A mice for ALS or other models for motor neuron diseases or hereditary myopathies as Marinesco–Sjögren Syndrome. MyoRep could also be subjected to partial or total body paralysis due to severe spinal cord injury or in the intensive care unit (ICU) model [34], or to mechanical local muscular trauma to follow atrophy. MyoRep could serve to unravel potential anti‐atrophic effects of lifestyle changes, for example, various dietary interventions and/or physical activity sessions of various aerobic or anaerobic exercise protocols.

The inability of MyoRep to sense circadian rhythm variations and its feature to hold transcription factor‐binding sites conserved across species, including humans, makes it suitable to generate clinical grade MyoRep‐AAV9 for early diagnostics in human patients, as done for other therapeutic AAV9 [35, 36]. We may consider treating MyoRep cells also with human fluids such as plasma or interstitial cancerous fluids or in the future aerosol‐derived samples from cancer patients. This would help to identify in advance patients at risk of cancer cachexia, for example, in order to intervene on time with proper countermeasures such as electrical muscle stimulation, exercise or nutritional supplementations [37], by sparing economic resources toward only those who may receive some real advantages. We are about to screen in vitro plasma from cancer patients to see if MyoRep‐expressing myoblasts and myotubes may predict in advance which patient will develop muscle atrophy.

MyoRep can sense more severe atrophy of cancer‐bearing males with respect to females at least in Apc^Min/+^ mice, helping us to understand sex‐specific mechanisms of atrophy to design novel therapies suited for sex, as we have shown for musclin. We have also reported the reversibility of the MyoRep induction by means of musclin‐AAV9 injections even if only locally administered. This indicates that such technology can be switched off by anti‐atrophic molecules, and that the MuRF1 promoter is at once a marker to follow atrophy, even in its early stages, and a way to see drug response. MyoRep mouse may serve eventually in the future to find effective drug doses, as we did in preliminary experiments not shown, to identify the right amount of AAV9 to administer.

Since *MuRF1* gene gets activated also in heart during its atrophy in the so‐called cardiac cachexia process [38] and given that the actin promoter of B6.Cg‐Tg(ACTA1‐cre)79Jme/J mice used to restrict the expression of MyoRep only in skeletal muscle is expressed also in the heart, we could follow in future studies MyoRep expression in the heart following various diseases. Cardiac MyoRep induction should be easily visualized because it is localized to a restricted region in the thorax. Nonetheless, we may plan to restrict MyoRep expression only in the heart by crossing the mouse with the stop codon with a mouse expressing Cre recombinase under a specific promoter for cardiac expression only (as the B6.FVB‐Tg (Myh6‐cre) 2182Mds/J mouse). The derived progeny may serve to understand mechanisms at the basis of heart failure obtained with coronary artery ligation or other microsurgeries in animal models [39].

### 4.3 Limitations of the MyoRep Technology

Despite having two reporter genes under the MyoRep promoter, *Firefly Luciferase* and *tdTomato*, we never visualized in optical microscopy in vivo neither in muscles dissected and analysed for their fluorescence ex vivo the expression of *tdTomato* for reasons that deserve further experiments. The second reporter gene is separated from the first one by IRES linker that perhaps allows too low expression of *tdTomato* to be detected. Another limitation of this tool is that *MuRF1* gene is not induced in all kinds of atrophy as that associated to microgravity in spaceflight [40] or in Duchenne muscular dystrophy (DMD) [41], making useless MyoRep to study these types of atrophy. Finally, the resolution of in vivo imaging is not enough to discriminate among different muscles, but only to identify grossly their position, and needs to be coupled with higher resolution imaging (microCT) or ex vivo analysis of separated muscles to better understand the origin of emitted signal in MyoRep mice. On a different note, current MyoRep mouse is on C57BL/6J background that while offering the advantage to cross it with many disease models, has the disadvantage that black hair can mask bioluminescence, so that we shaved the mice before imaging them. This problem could be circumvented by generating albino MyoRep mice or by systemically injecting MyoRep‐AAV9 in mice with white hair (i.e., BALB/c mice). Despite these limitations, we believe that MyoRep technology constitutes a real advancement to study in vivo muscle wasting because able to discriminate successfully between pathological and physiological atrophy.

## Ethics Statement

All animal studies have been approved by the appropriate ethics committee. The manuscript does not contain clinical studies or patient data.

## Conflicts of Interest

The authors declare no conflicts of interest, except for the Italian patent 102020000021598 (Sistema Reporter), owned by Università degli Studi di Milano and Fondazione Cariplo, which is directly related to the content of this publication. The inventors of MyoRep technology are R.P. (65%), N.R. (25%), A.M. (5%) and P.C. (5%). Some of the authors have additional current affiliations:

Martina Lunardi, Analytical Methods Development, AGC Biologics, Bresso, 20091, Italy; Mara Forti, TECHNOGENETICS S.p.a., Lodi, 26900, Italy and Giulia Terribile, School of Medicine and Surgery, University of Milano‐Bicocca, Monza, 20900, Italy.

## Supporting information


**Data S1:** Supporting information.

**Data S2:** Supporting information.

**Data S3:** Supporting information.

**Data S4:** Supporting information.

**Data S5:** Supporting information.

**Data S6:** Supporting information.

**Data S7:** Supporting information.

**Data S8:** Supporting information.

**Data S9:** Supporting information.

**Data S10:** Supporting information.

**Data S11:** Supporting information.

**Table S1:** Supporting information.

**Figure S1:** Tumour weight of MCG101 hosts sacrificed at different times. Kruskal–Wallis test followed by Dunn's post hoc test. ****p ≤ 0.0001. *N* = 7–33.
**Figure S2:** (A) GAS weight (% over PBS) of MCG101 hosts sacrificed at different times. Kruskal–Wallis test followed by Dunn's post hoc test. *p ≤ 0.05, **p ≤ 0.01. MCG101 20d vs. PBS, unpaired *t*‐test, **p ≤ 0.01. *N* = 7–33. (B) Luciferase assay analysis of TA from mice of Figure 2. Total proteins were used to normalize the data. *N* = 4–5. (C) Analysis of photon emission comparing the AUC of TA expressing TWIST vs. TA expressing GREDEL in PBS bearing‐mice. Unpaired *t*‐test for AUC, **p ≤ 0.01, *N* = 5.
**Figure S3:** GREDEL and TWIST plasmids electroporated in muscles in vivo were detectable in comparable amounts. Genomic DNA was extracted from the TA of mice electroporated with GREDEL or TWIST plasmids. LUC2 (A) and AMPi (Ampicillin Resistance) (B) inserts of plasmids were quantitated by qPCR to assess plasmid quantitation with ad hoc probes. Kruskal–Wallis test, *p < 0.05. As expected, LUC2 and AMPi detection were correlated (C). Spearman test, ****p < 0.0001.
**Figure S4:** Luciferase assays of C2C12 myoblasts transfected with GREDEL‐expressing plasmids and TK‐Renilla‐expressing ones (50:1) for 24 h and treated for various times with media conditioned by C26 or MCG101 cells or DMEM as control. Such treatment lasted for 6 (A), 24 (B) or 48 h (C). Protein content of C2C12 cells treated with conditioned media from C26 or MCG101 cells for 48 h was analysed by Bradford assay (D). One‐way ANOVA test followed by Dunnett's post hoc test. *p ≤ 0.05, **p ≤ 0.01, ***p ≤ 0.001, ****p ≤ 0.0001. All data are reported as mean ± SEM.
**Figure S5:** Cumulative food intake shown for MCG101‐ and PBS‐injected mice (cages = 1–2). Ns, multiple *t*‐test.
**Figure S6:** (A) A scheme for the generation of the MyoRep mouse. The stop sequence in Luc2 mouse is removed using the loxP system after crossing with the B6.Cg‐Tg(ACTA1‐cre)79Jme/J mouse. (B) Compared to the Luc2 mouse, the MyoRep mouse shows a basal bioluminescence signal, following the removal of the stop sequence.
**Figure S7:** The emission levels of MyoRep mice injected with PBS or the non‐cachectic MC38 tumour are comparable. Analysis of photon emission from the region of interest over time (ROI) of PBS (black line) and MC38 (red line) injected‐MyoRep mice acquired by in vivo imaging. Two‐way ANOVA, Tukey's post hoc test vs. Day 0, ns. *N* = 5. (A) Leg view, (B) dorsal view and (C) ventral view.
**Figure S8:** Ex vivo imaging of muscles and organs from MyoRep mice subjected to denervation (Den) or sham operation (Sham) after 35 days from cut of the sciatic nerve. Various muscles (TA, GAS for gastrocnemius, soleus and quadriceps) and various organs (spleen, brain, heart and liver) were freshly dissected by a representative mouse and subjected to ex vivo imaging. Luciferin was injected intraperitoneally about 20 min before this analysis.
**Figure S9:** Levels of musclin measured through ELISA in plasma of WT and Apc^Min/+^ male and female mice at 12, 15 and 18 weeks of age. WT includes samples of mice at the all indicated ages that have been pooled together. Kruskal–Wallis test followed by Dunn's post hoc test, **p ≤ 0.01. *N* = 10. All data are reported as mean ± SEM.
**Figure S10:** The alignment of transcription factor‐binding sites, boxed in red, is conserved across species and shown in the MyoRep promoter sequence. Analysis done with BLAST database.

## Data Availability

Raw data are shared by the corresponding author on request and deposited on Zenodo in the following link: https://doi.org/10.5281/zenodo.19335738. Further details to the MyoRep technology can be found here: https://www.knowledge‐share.eu/en/patents/reporter‐system‐for‐muscle‐atrophy and https://patentscope.wipo.int/search/en/detail.jsf?docId=WO2022054012&_cid=P11‐M73HPP‐93383‐1.
