# Supplementary material for: MyoRep: A Novel Reporter System to Detect Early Muscle Atrophy In Vitro and In Vivo
Source: J Cachexia Sarcopenia Muscle. 2026 May 12;17(3):e70296. doi: 10.1002/jcsm.70296 (PMC13167697; doi:10.1002/jcsm.70296)
Supplement: Supplementary file 10 — Data S10: Supporting information. [file JCSM-17-e70296-s001.pptx]

## Slide 1
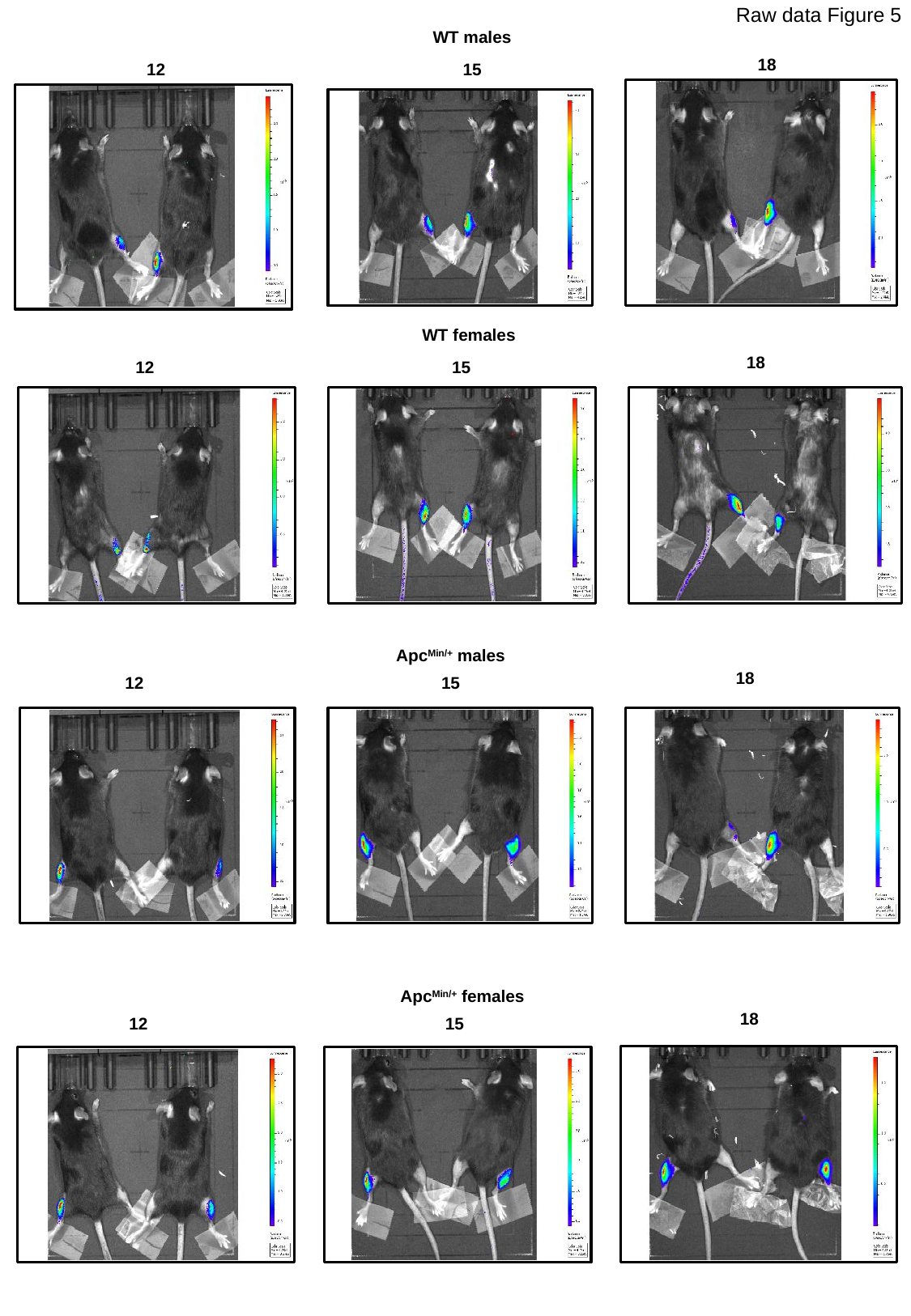

Raw data Figure 5
WT males
18
15
12
WT females
18
15
12
ApcMin/+ males
18
15
12
ApcMin/+ females
18
15
12

## Slide 2
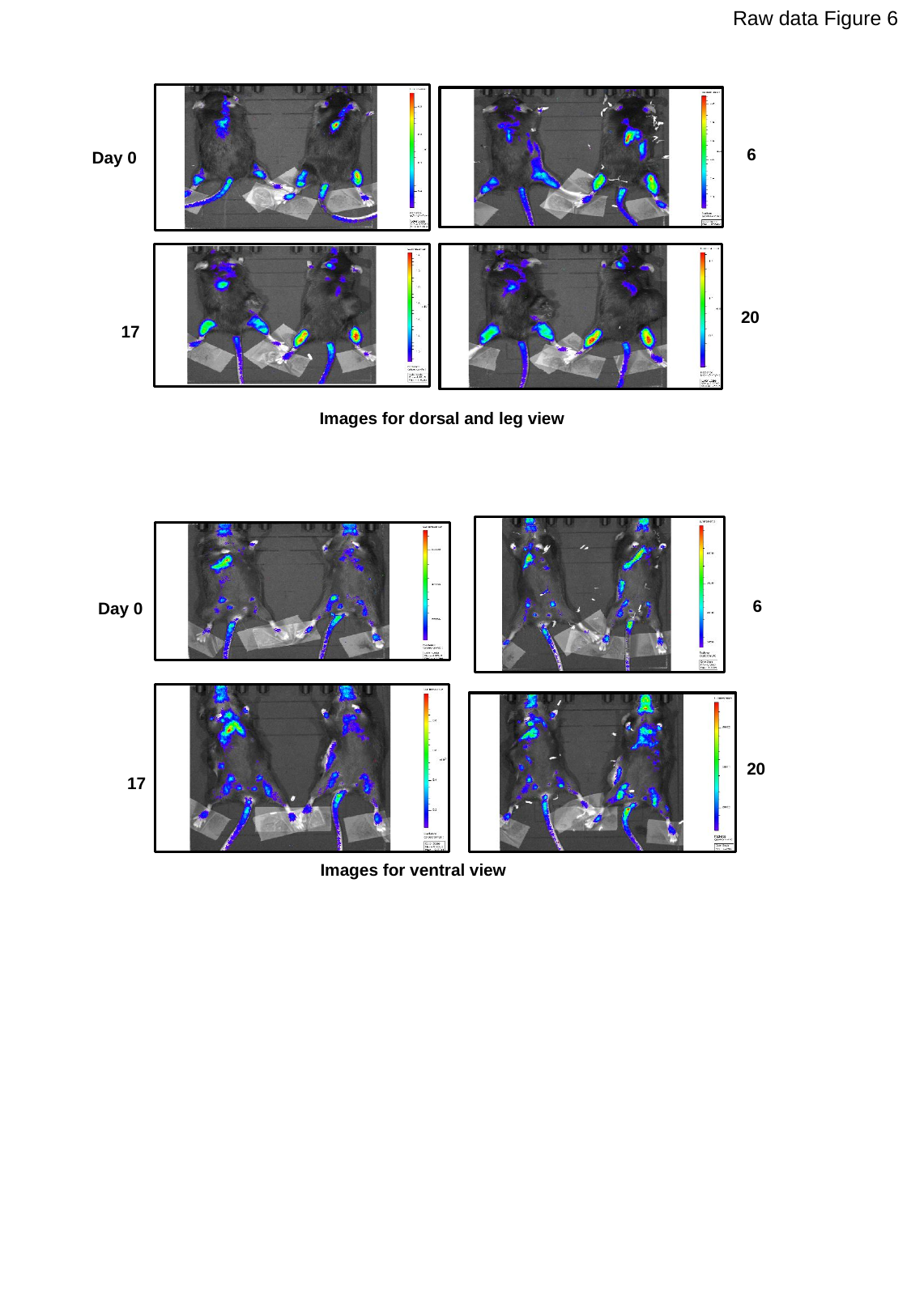

Raw data Figure 6
6
Day 0
20
17
Images for dorsal and leg view
6
Day 0
20
17
Images for ventral view

## Slide 3
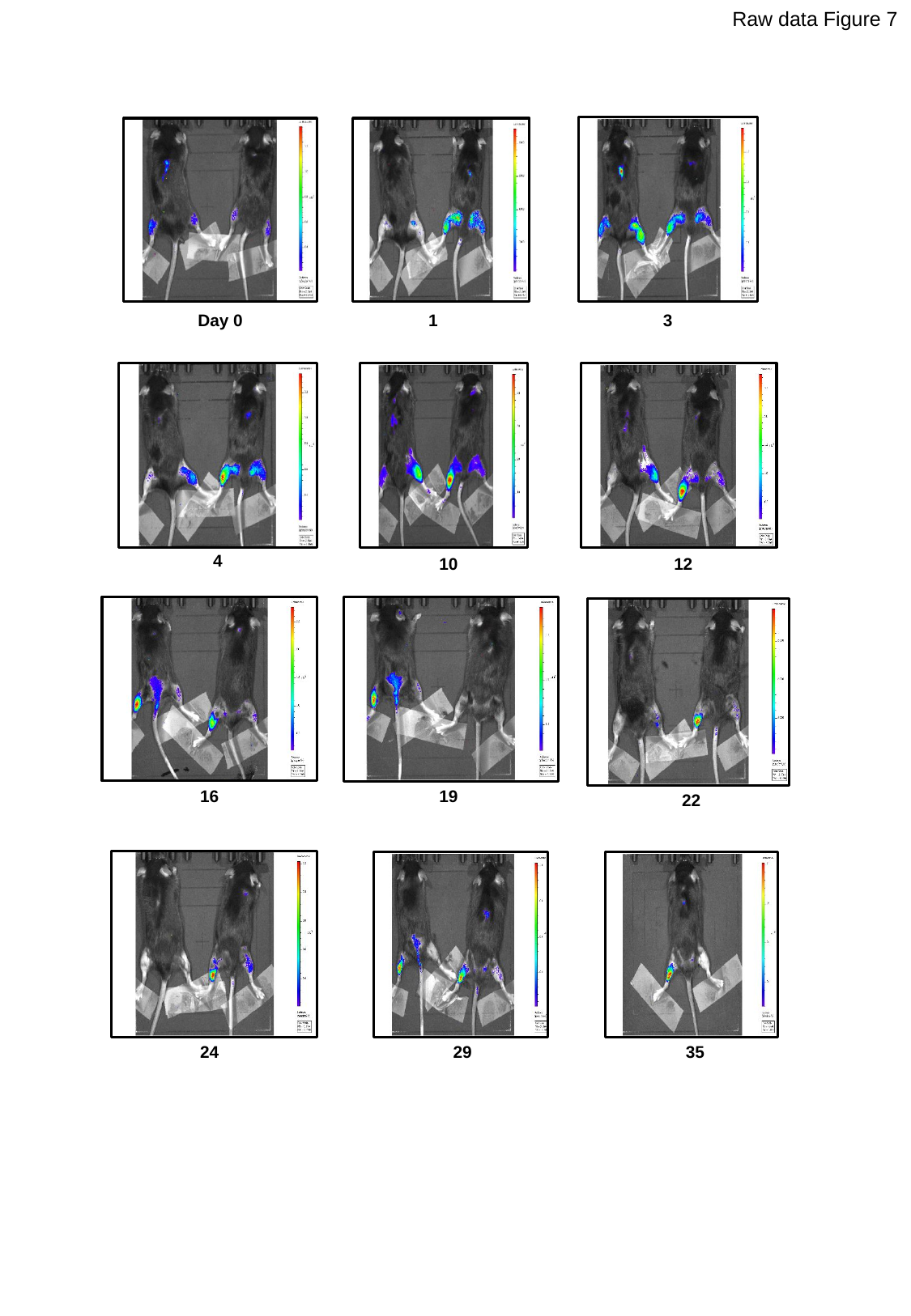

Raw data Figure 7
Day 0
1
3
4
10
12
19
16
22
24
29
35

## Slide 4
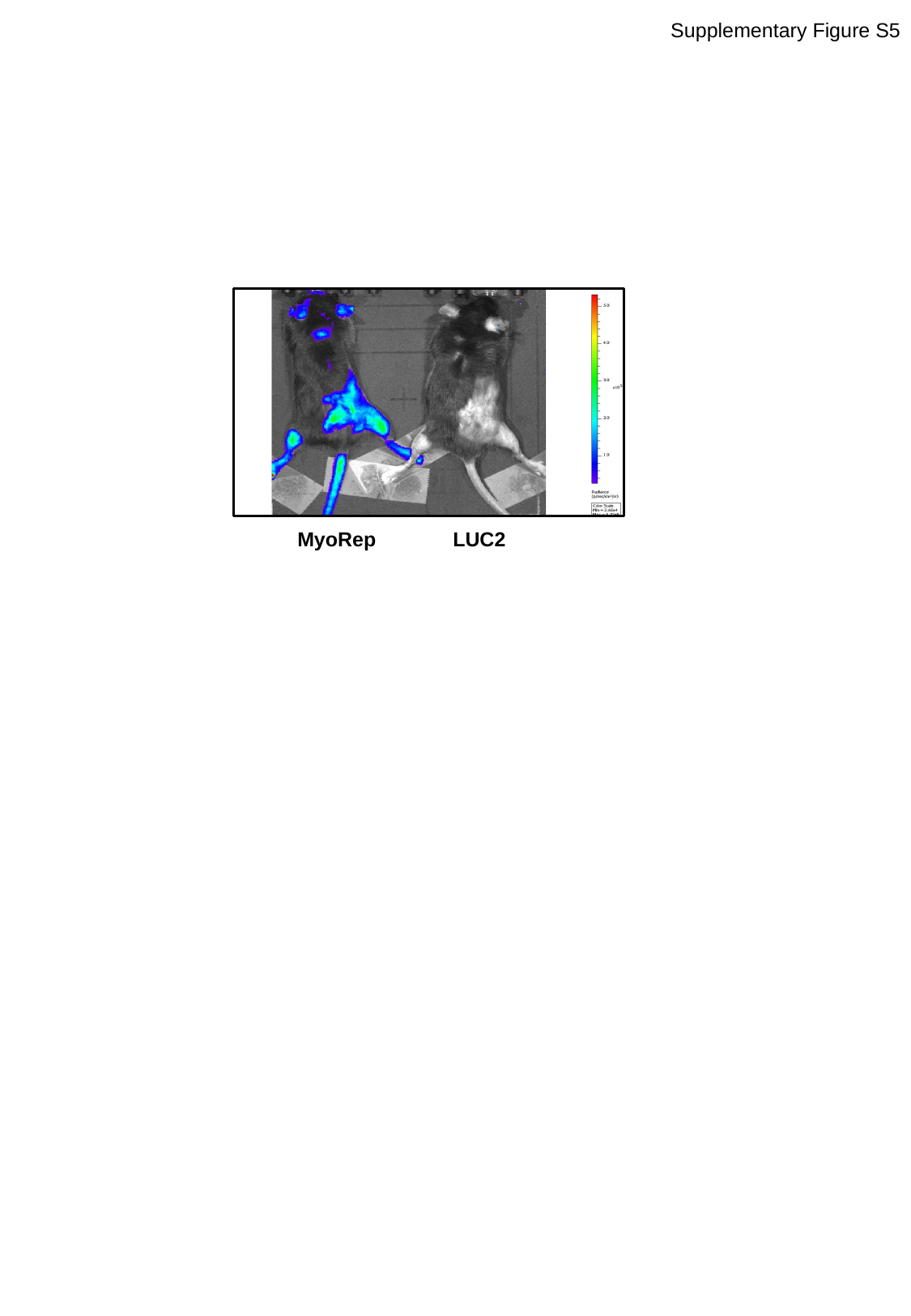

Supplementary Figure S5
MyoRep
LUC2
