# Supplementary material for: MyoRep: A Novel Reporter System to Detect Early Muscle Atrophy In Vitro and In Vivo
Source: J Cachexia Sarcopenia Muscle. 2026 May 12;17(3):e70296. doi: 10.1002/jcsm.70296 (PMC13167697; doi:10.1002/jcsm.70296)
Supplement: Supplementary file 11 — Data S11: Supporting information. [file JCSM-17-e70296-s012.pptx]

## Slide 1
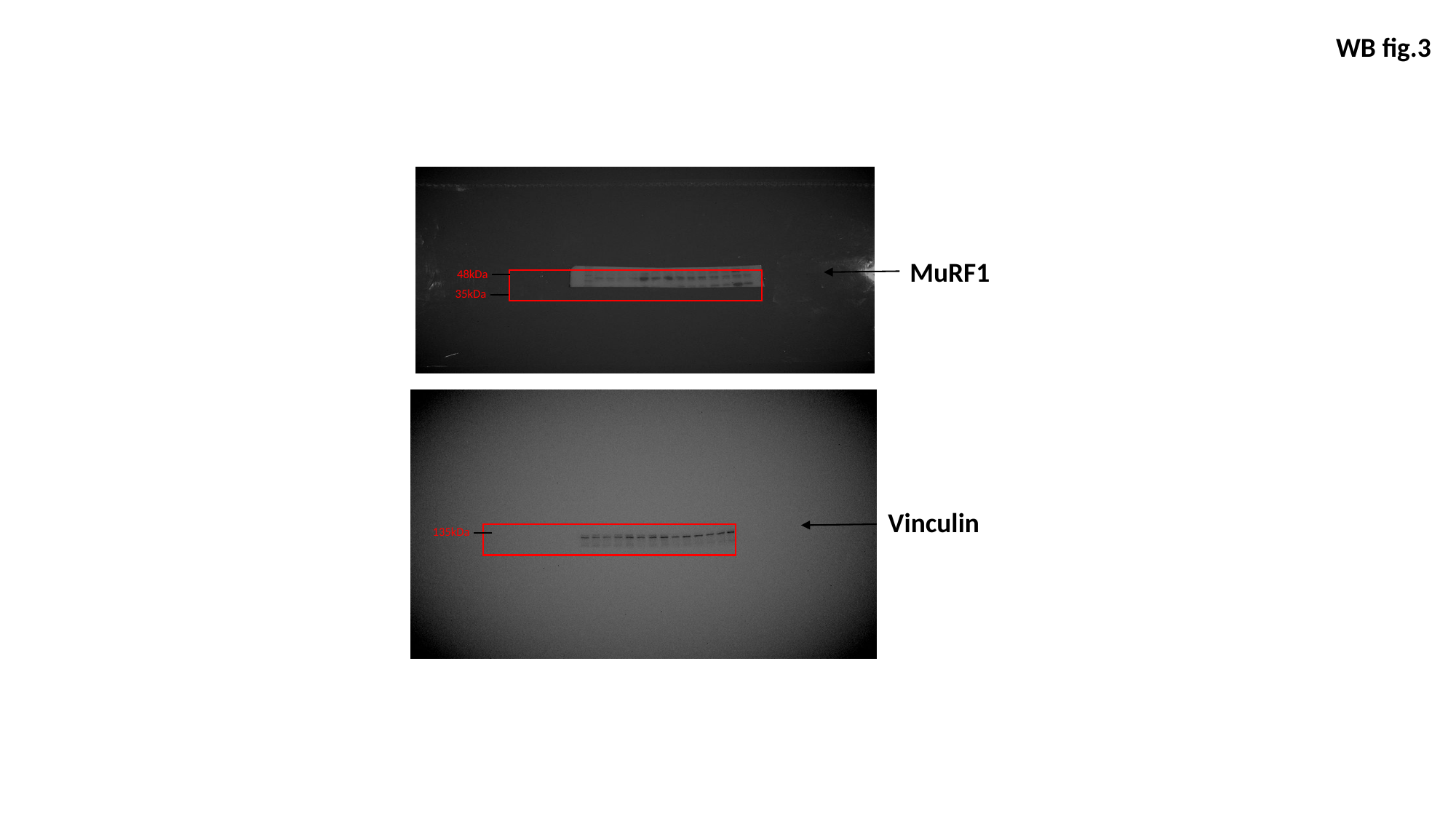

WB fig.3
MuRF1
48kDa
35kDa
Vinculin
135kDa

## Slide 2
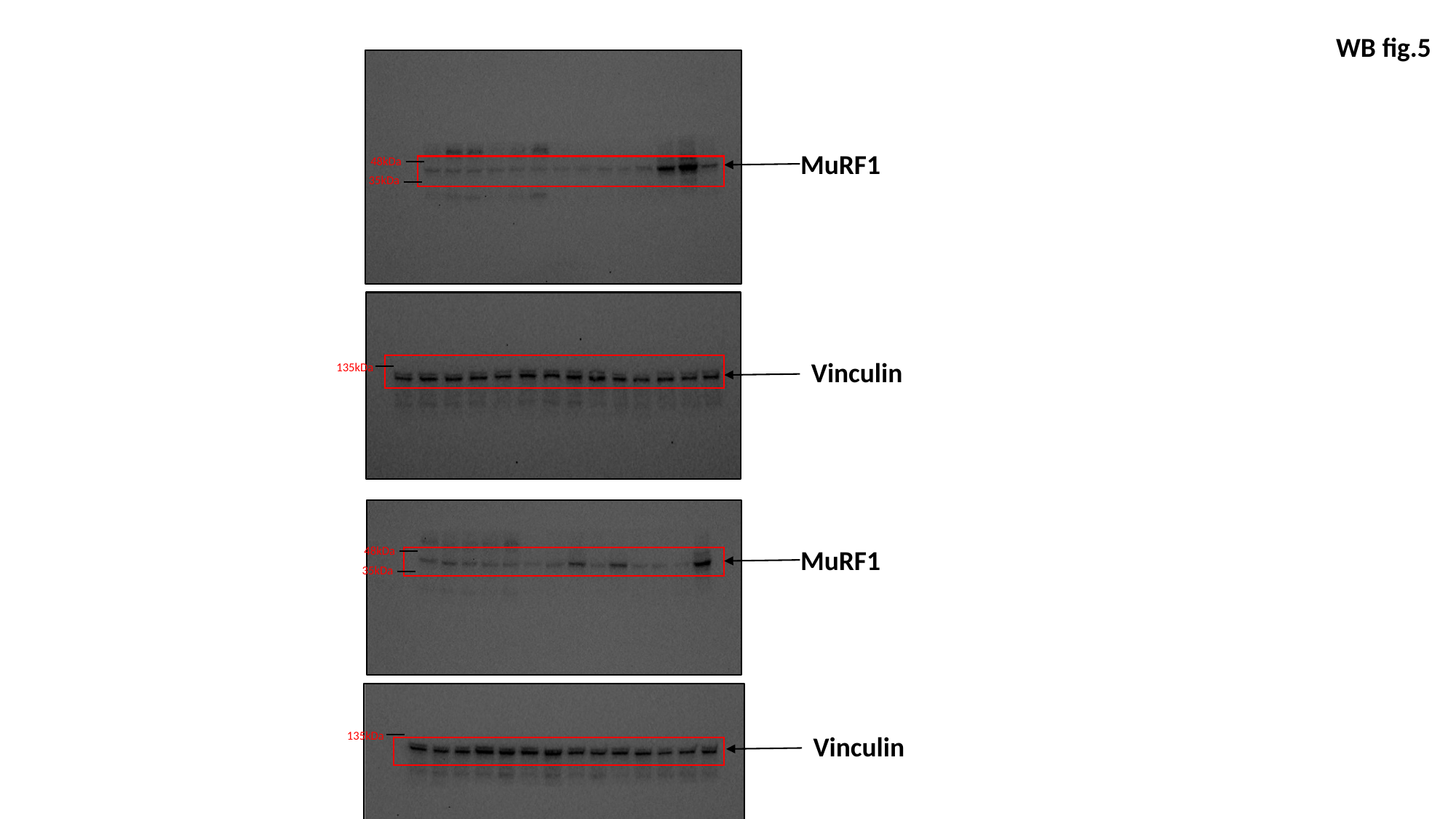

WB fig.5
MuRF1
48kDa
35kDa
Vinculin
135kDa
48kDa
MuRF1
35kDa
135kDa
Vinculin
