# Supplementary material for: MyoRep: A Novel Reporter System to Detect Early Muscle Atrophy In Vitro and In Vivo
Source: J Cachexia Sarcopenia Muscle. 2026 May 12;17(3):e70296. doi: 10.1002/jcsm.70296 (PMC13167697; doi:10.1002/jcsm.70296)
Supplement: Supplementary file 13 — Figure S1: Tumour weight of MCG101 hosts sacrificed at different times. Kruskal–Wallis test followed by Dunn's post hoc test. ****p ≤ 0.0001. N = 7–33. Figure S2: (A) GAS weight (% over PBS) of MCG101 hosts sacrificed at different times. Kruskal–Wallis test followed by Dunn's post hoc test. *p ≤ 0.05, **p ≤ 0.01. MCG101 20d vs. PBS, unpaired t‐test, **p ≤ 0.01. N = 7–33. (B) Luciferase assay analysis of TA from mice of Figure 2. Total proteins were used to normalize the data. N = 4–5. (C) Analysis of photon emission comparing the AUC of TA expressing TWIST vs. TA expressing GREDEL in PBS bearing‐mice. Unpaired t‐test for AUC, **p ≤ 0.01, N = 5. Figure S3: GREDEL and TWIST plasmids electroporated in muscles in vivo were detectable in comparable amounts. Genomic DNA was extracted from the TA of mice electroporated with GREDEL or TWIST plasmids. LUC2 (A) and AMPi (Ampicillin Resistance) (B) inserts of plasmids were quantitated by qPCR to assess plasmid quantitation with ad hoc probes. Kruskal–Wallis test, *p < 0.05. As expected, LUC2 and AMPi detection were correlated (C). Spearman test, ****p < 0.0001. Figure S4: Luciferase assays of C2C12 myoblasts transfected with GREDEL‐expressing plasmids and TK‐Renilla‐expressing ones (50:1) for 24 h and treated for various times with media conditioned by C26 or MCG101 cells or DMEM as control. Such treatment lasted for 6 (A), 24 (B) or 48 h (C). Protein content of C2C12 cells treated with conditioned media from C26 or MCG101 cells for 48 h was analysed by Bradford assay (D). One‐way ANOVA test followed by Dunnett's post hoc test. *p ≤ 0.05, **p ≤ 0.01, ***p ≤ 0.001, ****p ≤ 0.0001. All data are reported as mean ± SEM. Figure S5: Cumulative food intake shown for MCG101‐ and PBS‐injected mice (cages = 1–2). Ns, multiple t‐test. Figure S6: (A) A scheme for the generation of the MyoRep mouse. The stop sequence in Luc2 mouse is removed using the loxP system after crossing with the B6.Cg‐Tg(ACTA1‐cre)79Jme/J mouse. (B) Compared to the [file JCSM-17-e70296-s006.pdf]

SUPPLEMENTARY MATERIALS AND METHODS

Cell lines

C2C12 (ATCC, Manassas, VA, USA) is an immortalized mouse myoblast cell line obtained from the C3H mouse strain. It was grown in DMEM (Dulbecco's Modified Eagle's Medium, Gibco, Waltham, MA, USA), supplemented with fetal bovine serum (FBS) (Euroclone, Pero, Italy) and 2 mM L-glutamine, and maintained in culture at 37°C with 5% CO_2. C26 is a colorectal adenocarcinoma cell line obtained from BALB/c mice, grown in DMEM (Dulbecco's Modified Eagle's Medium, Gibco, Waltham, MA, USA), supplemented with 10% FBS and 2 mM L-glutamine at 37 °C with 5% CO_2. C26 cells were donated by Prof. Mario Paolo Colombo (IRCCS-Istituto Nazionale dei Tumori, Milan, Italy). MCG101 is a sarcoma cell line, grown in McCoy's 5A (Gibco) medium with 10% FBS and 2 mM L-glutamine at 37°C with 5% CO_2. MCG101 cells were shared by Prof. Anders Blomqvist (Linköping University, Linköping, Sweden). 4T1 cells were grown in Ham's F12 (Gibco) with 10% FBS and 2 mM L-glutamine, at 37°C with 5% CO_2. **MC38 cells were obtained by Dr. Silvia Guglietta from IEO, Milan, Italy. They grow in the same media as C2C12 cells.** All cell lines were not contaminated by mycoplasma.

Plasmids

The following plasmids are referred to the **Supplementary Table S1**:

- pGL3-FHRE ΔXRE, with 3 FoXO responsive elements without xenobiotic responsive ones, from Prof. Klotz, University of Alberta, Canada;
- PGL4.10 2D4F, with 4 FHRE and 2 DBE sequencing, engineered by us;
- pGL3-DBE (Daf-16 binding elements), with 6 Daf-16 binding elements, the ortholog of FOXO factors in *C. elegans*, from Prof. M. Sandri, University of Padova, Italy;
- pGL3-pAT1 3.5 Kb (Atrogin-1 promoter), from Prof. A.L. Goldberg, Harvard Medical School, Boston, USA;
- pGL3(CAGA)12 Firefly LUC (SMAD 2/3 binding sites), with 12 copies of (CAGA) box upstream of the minimal Adenovirus major late promoter (MLP) (TATA box + initiation sequence of MLP), it controls the expression of FLuc, from Prof. M. Sandri, University of Padova, Italy;
- pGL3 - 5 Kb MuRF1 promoter, from Prof. A.L. Goldberg, Harvard Medical School, Boston, USA;
- 4XM67-Luc-STAT3, with 4 STAT3 (M67) binding sites, from Prof. D. Bessel, Max-Delbrück-Centrum für Molekulare Medizin, Berlin, Germany;
- pNF-kβ Luc2-IRES-EGFP, as in [27];
- pGL4.30(Luc2P/NFAT-RE/Hygro), Promega, cat. n° E8481,

- TK-LXRE3-LUC, with 3 LXRE copies, from Prof. A. Moschetta, Università "Aldo Moro", Bari, Italy;
- pGL4.33(Luc2P/SRE/Hygro), Promega, cat. n° E1340;
- pGL4.29(Luc2P/CRE/Hygro), Promega, cat. n° E8471.

Cell treatments

C2C12 cells transfected with the above-mentioned plasmids were treated with conditioned medium of tumoral cells. Briefly, tumoral cells as C26, 4T1 and MCG101 were grown in their medium. After 48 hours, conditioned media were harvested when cells were 80-90% confluent, centrifuged for 5 minutes at 1,000 rpm. Such supernatant was then used to treat C2C12 for 24 hours. Transfected C2C12 cells were treated with dexamethasone for 24 hours, at the concentration of 1 and 10 μM.

***In vitro* and *ex vivo* Luciferase-based assays**

Luciferase assay was done on C2C12 cells grown in a 48 well-plate and transfected using Lipofectamine 2000 with different combination of plasmids, in the following relative proportions:

- mMuRF1-TWIST – pRL-TK-Renilla ratio 50:1;
- mMuRF1-GREDEL – pRL-TK-Renilla ratio 50:1;
- mMuRF1 – pRL-TK-Renilla ratio 50:1.

The next day, cells were treated with dexamethasone or media conditioned by tumoral cells. Firefly and Renilla luciferase activities were measured in cell lysates using the Dual-Luciferase Reporter Assay System (Promega, Madison, WI, USA) and the signal was quantitated using a luminometer (Glomax 20/20 single tube luminometer, Promega, Madison, WI, USA). Luciferase assay was also made *ex vivo*, on muscles excised from MyoRep mice inoculated with MCG101 tumor or C57BL/6J-Apc$^{Min/+}$ injected with MyoRep-AAV9 in Tibialis Anterior (TA) for 10 weeks. Muscles were lysed with Passive Lysis Buffer (PLB) and Firefly luciferase activity was measured using the Luciferase Assay kit (Promega). The signal was quantitated using the Glomax 20/20 single tube luminometer and related to total protein content of the sample measured with Bradford.

Total RNA extraction from muscles

Total RNA was isolated from muscles with QIAzol Lysis Reagent and miRNeasy Kit (Qiagen, Hilden, Germany). The frozen muscle was cut perpendicularly to the tendon and resuspended in 700 μl of QIAzol (Qiagen, Hilden, Germany). Muscles were then homogenized with T25 digital Ultra-Turrax homogenizer (IKA, Staufen, Germany). RNA was

extracted following manufacturer's instructions using chloroform and RNeasy Mini column provided by the kit. RNA concentration, purity and integrity were measured in a spectrophotometer (NANODROP 1000, ThermoFisher Scientific, Waltham, MA, USA).

Reverse transcription PCR

For the reverse transcription of RNA into cDNA, High-Capacity cDNA Reverse transcription Kit (Applied Biosystems, Waltham, MA, USA) was used. One µg of RNA was added to 4 µl of buffer 10X, 1,6 µl of dNTPs, 4 µl of random primers, 2 µl of reverse transcriptase enzyme and water reaching a final volume of 40 µl. Then, samples were placed in the thermocycler (BioRad T100 Thermal Cycler) at 25°C for 10 minutes, 37°C for 2 hours and 85°C for 5 minutes. To confirm the comparable amounts of reporter plasmids encoding for TWIST or GREDEL were electroporated into mouse legs, genomic DNA was isolated from the TA using the DNeasy Blood and Tissue Kit (Qiagen, Hilden, Germany) and then amplified by qPCR using the primers listed in the following table:

**Gene Symbol**	**Sequence**
AMPi R Forward	GACTCAAGGATCTTGCCGCT
AMPi R Reverse	TTTTTGCACACCCCGAAACG
Luc2 Forward	TTCGGCAACCAGATCATCCC
Luc2 Reverse	GTACATGAGCACGACCCGAA

Where AMPi R is a set of primers designed to amplify the *Ampicillin Resistance* gene present on the backbone of the vectors and Luc2 is a set of primers for *Firefly Luciferase* gene.

Quantitative Polymerase Chain Reaction

Analysis of mRNA in muscle was done using TaqMan reverse transcription reagents (Life Technologies Waltham, MA, USA) or the fluorescent intercalating DNA SYBR Green (Life Technologies Waltham, MA, USA). In each well of a Fast Optical 96 well-reaction plate, a mix containing TaqMan Master mix and the probe for TaqMan assay (Life Technologies Waltham, MA, USA) or SYBR Green Master mix and oligonucleotides as primers (Metabion, Planegg, Germany) were added to 2 µl of cDNA (20 ng). Water was added to reach a final volume of 11 µl. The instrument we used for these assays was a 7900HT Fast Real-Time PCR System (ThermoFisher Scientific, Waltham, MA, USA). The program for qPCR was as follows: step 1, 95°C for 15 min; step 2, 95°C for 25 sec; step 3, 60°C for 1 min; repeating

steps 2 and 3 for 40 cycles. *Gusb* (β-Glucuronidase) or *Tbp* (Tata binding protein) or *Ipo8* (Importin 8) were used as housekeeping genes. The following table contains the list of all primers used in this work.

Gene Symbol and Company	Forward Sequence	Reverse Sequence
Gusb, Metabion	TCGTACCAGCCACTATCCCTA	AAAACTCTGAGGTAGCACAATGC
TBP, Metabion	ACCCCACAACTCTTCCATTCT	TTTGAAGCTGCGGTACAATTC
Musclin, Metabion	GCATCACAGGAGTTTGGAACA	CAGATCATCAAGACGCAGGA
Ipo8, Life Technologies, Mm01255158_m1	Not available	Not available
Trim63/MuRF1 Life Technologies, Mm01185221_m1	Not available	Not available

Total protein extraction from tissues

Total proteins were extracted from muscles using radioimmunoprecipitation assay buffer (RIPA buffer) with the final addition of 4% sodium dodecyl sulphate (SDS), protease and phosphatase inhibitors (Roche, Basel, Switzerland). A volume of RIPA buffer corresponding to 20 times the weight of the muscle was added, then muscles were then homogenized with T25 digital Ultra-Turrax homogenizer. Each sample was vortexed for 1 minute, and then incubated for 40 minutes at 4°C on rotating wheel. For the last step, samples were centrifuged at 13,000 x g for 20 minutes at 4°C and the supernatant was collected and stored at -80°C.

Protein quantitation

The final protein concentration was quantitated by the bicinchoninic acid or BCA (Pierce, Waltham, MA, USA) or with Bradford if proteins were extracted with PLB from Luciferase assays. For BCA, each sample was diluted in water 1:10, and 10 µl were placed in a well of a flat transparent 96-well-plate, in duplicate. Then, 200 µl of BCA kit were added. On the same plate, known concentration of bovine serum albumin (BSA) were added. After 30 min of incubation at 37°C, using a spectrophotometer at 562 nm of wavelength, the absorbances were measured. The quantitation of the proteins was than extrapolated from the BSA standard curve based on the absorbance detected. For Bradford assay, each sample was diluted in water 1:10, and 10 µl were placed in a well of a flat transparent 96-well-plate, in duplicate. Then, 200 µl of Bradford substrate were added. On the same plate, known concentration of bovine serum albumin (BSA) were added. The absorbances were measured using a spectrophotometer at 595 nm of wavelength. Then, 10 µg of proteins were added

to Laemmli sample buffer 4X (Biorad, Hercules, CA, USA) supplemented with 10% β-mercaptoethanol (Sigma, St. Louis, MO, USA), and boiled at 97°C for 7 min.

Western Blotting

Each sample is placed into a well of a precast and gradient (4-20%) gel of sodium dodecyl sulfate polyacrylamide (BioRad) for electrophoresis (SDS-PAGE). The run was performed soaking the gels in running buffer (10% TGE, 1% SDS and H_2O) and applying at constant voltage (100 V) for 90 min. Later, proteins on gel were transferred to polyvinylidene difluoride membrane (PVDF; GE Healthcare, Chicago, IL, USA) using a transfer buffer (10% TGE, methanol and H_2O), for 1 hour at constant amperage (400 mA). Then, the membrane was incubated at RT with a 5% BSA solution (BSA – bovine serum albumin - dissolved in Tris-Buffered Saline-Tween (TBS-T) 1%). Then, the membrane was incubated overnight with a solution with 5% of BSA containing the primary antibody directed to the protein of interest. We used the following primary antibodies: Anti-MuRF1, kindly donated by Prof. Alfred L. Goldberg (Harvard Medical School, Boston, MA, USA) and anti-vinculin (V9264, Sigma). After the overnight incubation with the primary antibody and a 30 min wash with TBS-T, the membrane was incubated for 1 hour RT with the secondary antibody, diluted in a solution of 1% BSA in TBS-T and conjugated with alkaline phosphatase. The membrane was then washed with TBS-T for 30 minutes and, using CDP-star substrate (ThermoFisher Scientific, Waltham, MA, USA), proteins were detected by means of a chemiluminescence reaction, capture by the Odyssey Imager (Li-Cor, Bad Homburg, Germany). The intensity of the band was analyzed using the ImageJ software (National Institutes of Health, Bethesda, MA, USA).

Wheat germ agglutinin staining for CSA measurements

The cross-sectional area (CSA) shown was evaluated under blinded conditions using ImageJ software on 10-µm-thick cryosections of frozen TA muscles stained with wheat germ agglutinin (WGA) (ThermoFisher Scientific, Waltham, MA, USA). Images of muscle fibers were acquired using an Olympus Virtual Slide Microscope VS120 (Olympus, Shinjuku, Japan) equipped with a ×10 objective and ×10 ocular lens.

Enzyme-Linked Immunosorbent Assay

The levels of corticosteroids were measured in murine plasma of fed and fasted mice and PBS and 4T1-injected mice using an enzyme-linked immunosorbent assay (ELISA) kit (RTC002R, BioVendor, Brno, Czech Republic). The detection range of the kit is 11.4-819 ng/ml, depending on the sex of the mouse and the time of blood sampling. The lowest analytical detectable level of corticosterone is 6.1 ng/ml. Musclin levels in murine plasma were measured in an ELISA. Plasma was collected in 0.5 M EDTA, centrifuged 10 min at 10,000 rpm at 4°C, and then stored at −80°C.

Musclin was quantitated with a Cusabio ELISA kit (CSB-EL017269MO, Tema Ricerca, Castenaso, Italy). Diluted plasma samples and standards were applied to the ELISA plates, and incubated for 2 hours at 37 °C. Plates were then incubated with biotinylated antibody for 1 hour at 37°C. After three washes, plates were incubated with horseradish peroxidase (HRP)-avidin 1 hour at 37°C, then washed and incubated with the tetramethylbenzidine substrate for 15 min at 37 °C. Optical density was detected at 540 nm. The detection range of the kit is 15.6–1000 pg/mL and the sensitivity is about 3.9 pg/mL.

Muscle electroporation with plasmids

The Endotoxin-free Maxi Prep kit (Invitrogen, Carlsbad, CA, USA) was used to purify plasmids (TWIST and GREDEL) from bacteria for muscle electroporation. The animals were anesthetized by inhalation of 3% isoflurane and 1% O_2. Their hindlimbs were shaved and a linear incision of about 1 cm was made in the skin to expose the TA. One flatted electrode was then placed under the muscle, and using a 30-gauge Hamilton syringe, 20 µg of plasmid DNA in 30 ml of 0.9% NaCl solution in H_2O were injected in the muscle. The second flatted electrode was then placed over the muscle, which was electroporated with five pulses (21 V) of 20 milliseconds (ms) each, with a 200 ms interval. Electroporation was done with the BTX ECM 830 Square Wave Electroporation System (Harvard Apparatus, Cambridge, MA, USA). Finally, the wound was sutured and disinfected with betadine.

MyoRep-AAV9

Adeno-associated viruses 9 (AAV9) that express the MyoRep construct or musclin were injected intramuscularly into TA of $Apc^{Min/+}$ mice or WT mice at 10 weeks of age of both sexes the. Based on our preliminary data not shown, we set out to use the concentration of 10^{12} vg/ml of such AAV9 in 30 µl of PBS per leg of mouse. Recombinant AAV9 vectors used in this study were prepared by the AAV Vector Unit at the International Centre for Genetic Engineering and Biotechnology Trieste. Briefly, infectious AAV vector particles were generated in HEK293T cells cultured in roller bottles by a three-plasmids transfection cross-packaging approach whereby the vector genome was packaged into AAV capsid serotype-9. Purification of viral particles was obtained by PEG precipitation and two subsequent CsCl gradient centrifugations. The physical titer of recombinant AAVs was determined by absolute quantification of vector genomes (vg) packaged into viral particles, by qPCR.

***In vivo* imaging**

WT mice after TWIST and GREDEL electroporation or AAV9 injection in TA and MyoRep mice emit a bioluminescent signal that can be easily detected through *in vivo* imaging (IVIS machine, Perkin Elmer, Milan, Italy).

Averagely once a week, we performed an IVIS scan. We injected a volume of luciferin (Perkin Elmer – 20 mg/kg) and 5 minutes later, we anesthetized mice in the induction chamber, shaved the hind legs, as the hair shields the bioluminescence, and we placed them into the instrument, in dorsal position, exposing the TA and GAS muscles of both legs as best as possible, or in ventral position. Ten minutes after the luciferin injection, we performed one minute-scan for bioluminescence (for plasmids electroporation and AAV9 injection) and 5 minutes-scan for ventral and dorsal views (for MyoRep mice). The analyses were made with the Living Image Software (Perkin Elmer).

Mouse fasting and refeeding

Mice were subjected to fasting for either 16 or 48 hours. Fasted animals were transferred to new, clean cages without food but with free access to water, while maintaining environmental enrichment. After 48 hours of fasting, food was reintroduced. *In vivo* imaging was performed 16 or 48 hours after food deprivation and 24 hours after refeeding.

Unilateral cut of the sciatic nerve of mice

Cutting the sciatic nerve was done on WT mice to induce atrophy of the hindlimb muscles. Anesthetized mice were placed in a prone position and a small incision was made on the thigh to expose the sciatic nerve. The nerve was isolated using tweezers, and a 2-3 mm segment was excised to prevent reinnervation. The incision was closed with absorbable sutures and disinfected with betadine. Sham-operated mice underwent the same procedure without nerve cutting.

Mouse sacrifice and tissue collection

Mice have been euthanized by decapitation when sleeping because of exposure to gas anesthesia with a mixture of 100% oxygen (2 l/min) and 5% isoflurane. Mice have been sacrificed when they reached a BWL higher than 20% for 3 consecutive days or when the tumor reached 10% of total BW (and before ulceration) or when four out of five of the following parameters were present in the same moment: immobility, hypothermia, kyphosis, tremor and ruffled coat. $Apc^{Min/+}$ mice have been sacrificed through similar criteria, but also considering different time points (15 and 18 weeks of age). We collected blood to prepare plasma, hindlimb muscles (TA, GAS, soleus, quadriceps, back muscles). For plasma, blood was collected from mice in BD Microtainer additive K2-EDTA beadless cap (BD, Milan, Italy), tubes that contain EDTA as anticoagulant, and centrifuged for 5 minutes at 5,000 x g at 4°C. Once dissected, muscles and organs have been weighed with analytical scale (Bel engineering srl, Monza, Italy) and frozen in liquid nitrogen-cooled isopentane (-190°C) or analyzed for *ex vivo* imaging with IVIS system.

C57BL/6J-Apc$^{Min/+}$ mouse model

In C57BL/6J-Apc$^{Min/+}$ (Apc$^{Min/+}$) mice, colorectal cancer-induced cachexia is evident around 12-14 weeks of age and their average lifespan is about 20-25 weeks. To genotype them, we proceeded with a PCR using a little biopsy from the mouse ear, using the primers in the following **Table I**:

Gene Symbol	Sequence
APC – WT	GCCATCCCTTCACGTTAG
APC – Common	TTCCACTTTGGCATAAGGC
APC – Mutant	TTCTGAGAAAGACAGAAGTTA

Apc$^{Min/+}$ mice have been weighted once a week starting from 9-10 weeks of age, then, when the first signs of cachexia appear, later 3 times a week, until euthanasia.

MCG101, MC38 and 4T1 mouse models

MCG101 cells were seeded at a density of 12,000 cells/cm^2, **while MC38 at 17,000 cells/cm^2** and 4T1 cells at 25,000 cells/cm^2 in a filtered-capped flask. Forty-eight hours later, cells were detached using trypsin-EDTA 0.25% (Gibco, Waltham, MA, USA) and counted through Beckman Coulter Counter. Then, they were centrifuged for 10 minutes and the pellet was resuspended in sterile PBS, to obtain 0,5 × 10^6 cells in 200 µl of sterile PBS (phosphate buffered saline) for MCG101, **10^6 cells for MC38** and 2 × 10^5 for 4T1 for each mouse. MCG101 **or MC38** cells were subcutaneously injected into the upper right flank of C57BL/6J or C57BL/6J-MyoRep male mice of 10-12 weeks of age using a 1 ml syringe with a 25 Gauge-needle. 4T1 cells were subcutaneously injected into the upper right flank of BALB/c male mice of 10-12 weeks of age (Envigo, Indianapolis, IN, USA) using a 1 ml syringe with a 25 Gauge-needle. The control groups received 200 ml of sterile PBS.

MyoRep reporter mouse

In the MyoRep plasmid the promoter was cloned into the NF-kβ-loxP-STOP1x-loxP-luc2-ires-tdTomato plasmid by substituting the NF-kβ responsive promoter with MyoRep promoter in the XhoI/AscI restriction sites by using standard cloning procedures. In this way, we obtained pTargeting-MyoRep [27]. To identify the best promoter sequences and responsive elements we analyzed a panel of atrogenes, paying attention to the conserved responsive elements included in the majority of atrogenes and also the sequences of responsive elements typical of the early atrogenes. At the end of such bioinformatics analysis, the sequence identified as the best one to do MyoRep system was derived from MuRF1 promoter, but we deleted some regions and dimerized a short fragment that holds several well-conserved binding sites: E-Boxes

(EM1, EM2 and EM3) binding sites for Twist; FOXO-, Smad- and myogenin-binding sites, as indicated in the sequences below:

gggtggggcggagaccaacttctgcaggaaactagggtatctggctctcccctgaacctggctc**ttgttta**cgaccccca<u>cggcagggcaa**cagcga**ttg</u>ctcatcgggtg gggcggagaccaacttctgcaggaaactagggtatctggctctcccctgaacctggctcttc

ttgtttacgaccccca cggcagggcaa**cagcga**ttgctcatcccctgccagcagcctggctgcggaatgct**cagctg**gtcccctcctggggctcatgtgacagaggtgcag

c**<u>tataaata</u>**tcagaggggcct**cagac**caagac

1 smad **cagac**

2 myogenin-binding elements **cagcga**

2 foxo-binding elements **ttgttta**

1 tata e foxo1 **<u>tataaata</u>**

2 EM1 cggagaccaacttctgcagg

<u>2 EM2 cggcagggcaa**cagcga**ttg</u>

<u>1 EM3</u> **ctg**gtcccctcctggggctc

The targeting vector MyoRep was linearized with NotI and transferred into sv6.4 embryonic stem cells by electroporation: 35 µg/DNA each using 15 million cells (Core Facility for Conditional Mutagenesis, DIBIT San Raffaele, Milan, Italy). Positive clones were selected with puromycin (1 µg/µl). More than four hundred resistant clones for each transgene were screened for homologous recombination by PCR. One positive clone was injected into C57BL/6NCrL blastocysts which are transferred to pseudo-pregnant CD-1 females. We obtained 9 chimeric male mice (with 80–90% of chimerism) that were mated to wild-type (WT) C57BL/6J female mice to produce F1 MyoRep-stop transgenic mice. The stop sequence in MyoRep-stop (Luc2) mouse was removed using the loxP system after crossing with the B6.Cg-Tg(ACTA1-cre)79Jme/J mouse (The Jackson Laboratories, USA). Compared to the Luc2 mouse, the MyoRep mouse shows a basal bioluminescence signal following the removal of the stop sequence (**Supplementary Figure S5**).

To genotype CRE and Luc2 mice, we performed a PCR on a little biopsy from the mouse ear, using the primers in the following **Table II**:

Gene Symbol	Sequence
CRE Forward	AATGCTCTGTCCGTTTGCCGGT
CRE Reverse	CCAGGCTAAGTGCCTTCTCTACA
Luc2 Forward	GATCAAGTAGCCCAGCGTGGTG
Luc2 Reverse	AGCCCACCGTCGTATTCGTGA

Instead, the sequence of the so-called mMuRF1-TWIST promoter is as follows:

gggtggggcggagaccaacttctgcaggaaactagggtatctggctctccctgaacctggctc**tgaacagtctgttcttgttta**cgacccccacggcagggcaa**cagcg**

attgctcatcgggtggggcggagaccaacttctgcaggaaactagggtatctggctctccctgaacctggctc**tgaacagtctgttcttgttta**cgacccccacggcaggg

caa**cagcga**ttgctcatccctgccagcagcctggctgcggaatgctcagctggtcccctcctggggctcatgtgacagaggtgcagc**tataaata**tcagaggggcc**t**cag

ac**caagac**

1 smad cagac

2 myogenin-binding elements **cagcga**

2 foxo-binding elements **ttgttta**

1 tata e foxo1 **tataaata**

2 EM1 cggagaccaacttctgcagg

2 EM2 cggcagggcaa**cagcga**ttg

1 EM3 **ctgg**tcccctcctggggct

2 Glucocorticoid-responsive elements

Finally, the sequence of the so-called mMuRF1 promoter is as follows:

gcaagcccctttaccactgagccatcctgctggccctcaagaaagccttgccagcctaagttcaatgactggctgagggctgtcagggtgggctgcccaacatggagtctcaa

aagactctactgaaatgcttgactggagtctgaggcaggagaaagggctcacagaaagggctatcccaaggccttagagctgttcagaatccaggaggggaacagatttagt

ttgatacaggagtgctggtcttaaatgccagccccaaccccgcacccccacccccacccccaaacccaggttccattcccttactcccaattgactggagggggcggggatgg

ggcaggggcggggagtgggggtggggcggagaccaacttctgcaggaaactagggtatctggctctcccctgaacctggctc**tgaacagtctgttcttgttta**cgacccc

cacggcagggcaacagcgattgctcatccctgcatgtgatctgagagggccaaatctttgaggcctggaggaaactcaagccctgccagcagcctggctgcggaatgctca

gctggtcccctcctggggctcatgtgacagaggtgcagctataaatatcagaggggcctcagaccaagac
